# Near full-length 16S rRNA gene next-generation sequencing revealed *Asaia* as a common midgut bacterium of wild and domesticated Queensland fruit fly larvae

**DOI:** 10.1186/s40168-018-0463-y

**Published:** 2018-05-05

**Authors:** Ania T. Deutscher, Catherine M. Burke, Aaron E. Darling, Markus Riegler, Olivia L. Reynolds, Toni A. Chapman

**Affiliations:** 1 Present Address: Biosecurity and Food Safety, NSW Department of Primary Industries, Elizabeth Macarthur Agricultural Institute, Menangle, NSW Australia; 2 Graham Centre for Agricultural Innovation (an alliance between NSW Department of Primary Industries and Charles Sturt University), Elizabeth Macarthur Agricultural Institute, Menangle, NSW Australia; 3 The ithree institute, University of Technology Sydney, Sydney, NSW Australia; 4 School of Life Sciences, University of Technology Sydney, Sydney, NSW Australia; 5 Hawkesbury Institute for the Environment, Western Sydney University, Richmond, NSW Australia

**Keywords:** *Diptera*, *Tephritidae*, *Bactrocera tryoni*, Sterile insect technique, Host–microbe interactions, Insect–microbe interactions, Illumina, Microbiota, Microbial symbiont, Acetic acid bacteria

## Abstract

**Background:**

Gut microbiota affects tephritid (*Diptera: Tephritidae*) fruit fly development, physiology, behavior, and thus the quality of flies mass-reared for the sterile insect technique (SIT), a target-specific, sustainable, environmentally benign form of pest management. The Queensland fruit fly, *Bactrocera tryoni* (*Tephritidae*), is a significant horticultural pest in Australia and can be managed with SIT. Little is known about the impacts that laboratory-adaptation (domestication) and mass-rearing have on the tephritid larval gut microbiome. Read lengths of previous fruit fly next-generation sequencing (NGS) studies have limited the resolution of microbiome studies, and the diversity within populations is often overlooked. In this study, we used a new near full-length (> 1300 nt) 16S rRNA gene amplicon NGS approach to characterize gut bacterial communities of individual *B. tryoni* larvae from two field populations (developing in peaches) and three domesticated populations (mass- or laboratory-reared on artificial diets).

**Results:**

Near full-length 16S rRNA gene sequences were obtained for 56 *B. tryoni* larvae. OTU clustering at 99% similarity revealed that gut bacterial diversity was low and significantly lower in domesticated larvae. Bacteria commonly associated with fruit (*Acetobacteraceae*, *Enterobacteriaceae*, and *Leuconostocaceae*) were detected in wild larvae, but were largely absent from domesticated larvae. However, *Asaia,* an acetic acid bacterium not frequently detected within adult tephritid species, was detected in larvae of both wild and domesticated populations (55 out of 56 larval gut samples). Larvae from the same single peach shared a similar gut bacterial profile, whereas larvae from different peaches collected from the same tree had different gut bacterial profiles. Clustering of the *Asaia* near full-length sequences at 100% similarity showed that the wild flies from different locations had different *Asaia* strains.

**Conclusions:**

Variation in the gut bacterial communities of *B. tryoni* larvae depends on diet, domestication, and horizontal acquisition. Bacterial variation in wild larvae suggests that more than one bacterial species can perform the same functional role; however, *Asaia* could be an important gut bacterium in larvae and warrants further study. A greater understanding of the functions of the bacteria detected in larvae could lead to increased fly quality and performance as part of the SIT.

**Electronic supplementary material:**

The online version of this article (10.1186/s40168-018-0463-y) contains supplementary material, which is available to authorized users.

## Background

Globally, some of the most economically damaging horticultural pests are fruit flies of the family *Tephritidae* (*Diptera*). In Australia, the Queensland fruit fly, *Bactrocera tryoni* (*Tephritidae*), is a significant economic pest that causes losses due to damage of horticultural produce, while also restricting domestic and international market access [1]. *Bactrocera tryoni* is highly polyphagous, with females ovipositing and larvae developing in a large range of host fruits and fruiting vegetables [2–4]. The larvae and bacteria introduced into the fruit during oviposition cause the fruit to rapidly decay [5]. Although *B. tryoni* originated from tropical and subtropical regions of Australia, its range has expanded further down the east coast of Australia with increasing detections further inland. It is now established in New South Wales and Victoria, apart from the Greater Sunraysia Pest-Free Area [6]. Outbreaks also occur infrequently in South Australia and Western Australia [6, 7]. Severe restrictions or withdrawal of two of the most effective agrochemicals against *B. tryoni*, fenthion and dimethoate, requires alternative control options to be available to growers [8, 9]. The sterile insect technique (SIT) is a species-specific, sustainable, environmentally benign pest management strategy that involves mass-rearing of the target insects. The insects are irradiated to render them sterile prior to release in the field, where the sterile males copulate with wild females of the target pest population and are unable to produce viable offspring [10]. Over time and with repeated releases of sterile flies, the pest population is suppressed or may be eradicated within the release area.

The quality and performance of the released sterile insects are critical to the cost efficiency and efficacy of SIT programs. By impacting on host nutrition, physiology, metabolism, and immunity, gut microbiota can profoundly influence various aspects of insect health, fitness, and behavior [11, 12]. As a result, changes in gut microbiota associated with fruit fly domestication, mass-rearing, and sterilization are also likely to impact the quality of fruit flies produced for SIT programs.

Fruit flies reared under artificial conditions are no longer exposed to the microorganisms present in their natural environment. In addition, artificial larval diets typically contain antimicrobials and have a low pH to control bacterial and fungal contamination [13]. Consequently, laboratory- or mass-reared adult fruit flies (including *Drosophila*) are reported to have decreased gut microbial diversity and density compared to wild flies [14–16]. Shifts in bacterial types and relative abundances present in adult flies have also been reported between laboratory and wild flies [17–19]. The addition of bacteria from the *Enterobacteriaceae* family to the larval diets used to rear the Vienna 8 genetic sexing strain (GSS) of *Ceratitis capitata* (*Tephritidae*) used in SIT programs significantly reduced fly developmental time (egg to pupae and egg to adult), but not at the expense of fly quality [20]. It also increased pupal weight, longevity, morphometric traits (greater head width, abdomen, and thorax length), mating competitiveness under laboratory conditions, and spermatozoa storage in females [21]. Therefore, addition of probiotic bacteria may be a means of restoring or improving gut bacteria to positively influence fly production and performance.

To appreciate how insect–microbe interactions can impact the quality and competitiveness of mass-reared *B. tryoni* for SIT programs, it is necessary to determine how the gut bacterial diversity and community composition of laboratory- and mass-reared *B. tryoni* differs to that of wild flies. Knowledge of the diversity within populations is also required to identify “core” bacteria and provide context to the differences in gut microbial communities between populations. Previously, next-generation sequencing (NGS) was employed to identify the microbiome of adult domesticated and wild *B. tryoni* populations on a 454 pyrosequencing platform [19]. The study, by Morrow et al. (2015), detected shifts in the types of bacteria present and their relative abundance between laboratory-reared flies and their wild counterparts, but only analyzed the bacterial communities of whole adult female flies and did not look at the diversity in larvae and between individuals within populations. Pooling of samples and analysis of only a small number of sample pools, as well as the NGS of short amplicons, limited the ability to characterize the relative abundance of taxa and the identification of core bacteria across *B. tryoni* individuals.

Very few “core” bacterial species have been identified in tephritids. Unculturable “*Candidatus* Erwinia dacicola” is an important symbiont of olive fly, *Bactrocera oleae* (*Tephritidae*), in field populations, as it helps the larvae overcome plant chemical defense mechanisms and develop in unripe olives [14]. *Acetobacter tropicalis* was reported as highly prevalent and part of the “core” microbiota of *B. oleae* in one study [22], but was not detected in other recent studies [14, 23]. Identifying other important, possible “core” symbionts of fruit flies has been limited due to studies not reporting on bacterial community variation within fruit fly populations as a result of analyzing either only a single sample, pooling samples, or only a very small total number of individual samples from a limited number of populations. At a higher level, *Enterobacteriaceae* are found in the majority of published tephritid studies that mostly investigated adult individuals [5, 19, 24–27], are reportedly transferred vertically in some species [24, 28–31], and thus are considered important for tephritid development and physiology. *Enterobacteriaceae* are the prevalent bacterial family in wild adult *B. tryoni* populations [19, 25, 27, 32]. Sequencing only short regions greatly reduces the accuracy or reliability in characterizing taxa for a large number of bacteria at the genus level, especially for *Enterobacteriaceae* [24]. Further, the majority of insect bacterial community studies using NGS have only sequenced short fragments of the 16S rRNA gene, which can over- or under-estimate bacterial diversity [33, 34]. Various *Pseudomonadaceae*, *Acetobacteraceae*, and *Lactobacillales* have also been detected in several tephritids, but unlike in *Drosophila* where *Lactobacillus* spp. and *Acetobacter* spp. are common genera [35], very little attention has been paid to these bacterial families in tephritids, apart from *A. tropicalis* (*Acetobacteraceae*) in *B. oleae*.

The adult fly has been the focus of the majority of tephritid gut microbiota studies. Since larval nutrition in holometabolous insects can influence adult morphology and fitness [36–40] and insect symbiotic microbes can also influence host nutrition [41, 42], it is important to identify gut bacterial composition and diversity of larvae in addition to adults. Gut microbiota are not only a source of nutrients but can also assist the host insect in nutrient acquisition, allocation, assimilation, and detoxification [14, 23, 41, 43, 44]. For example, “*Ca.* E. dacicola” plays a detoxification role in *B. oleae* larvae [14]. In contrast to the many studies on the microbiome of adult tephritids, only few studies have studied the larval microbiome of tephritids, and no previous study has investigated the bacterial communities that constitute the larval gut of *B. tryoni*.

In this study, we characterize the gut bacterial diversity and community composition of individual domesticated and wild *B. tryoni* larvae using the near full-length 16S rRNA gene amplicon NGS method, a sequencing method recently developed by Burke and Darling [45]. In addition to offering finer phylogenetic resolution, which we deemed important for our aim of identifying probiotic candidates, as it could be a species rather than family of bacteria that was present/absent in wild versus domesticated larvae, the dual-end tagging of single 16S rRNA molecules in this method also increases the ability to detect and exclude chimeras [45]. Previously, this method was employed to analyze human skin microbial communities and showed that the taxonomic profiles were shown to be highly concordant with V4 region of the 16S rRNA gene amplicon sequencing of the same communities and offer higher taxonomic resolution [45]. This is the first time this methodology has been applied to an ecological study. We show that domestication and mass-rearing reduce the gut microbial diversity of larvae and identify both similarities and differences in the structure of the bacterial communities of larvae from wild and artificial rearing environments. This study highlights the need to understand the impact of changes in gut microbial species and how the loss of microbial diversity during domestication may influence fly performance in SIT programs.

## Methods

### Collection, identification, and dissection of *B. tryoni* larvae

To characterize the gut bacterial communities of *B. tryoni* larvae from wild populations, *B. tryoni*-infested white-fleshed peaches were sourced from one tree, at each of two locations, Buxton (Bux.P) and Tumut (Tum.P), in New South Wales, Australia (Table 1). While more peaches were collected, midgut bacteria of larvae from seven peaches (peaches A-G) from Buxton and five peaches from Tumut (peaches A-E) were analyzed. Individual larvae from the respective single peach were numbered 1 to 3; therefore, larvae from Buxton peach A were called Bux.P.A1, Bux.P.A2, and Bux.P.A3. *Bactrocera tryoni* is one of a number of species that belong to the *B. tryoni* species complex, which are difficult to distinguish using molecular methods [46]. Of this species complex, generally *B. tryoni* and occasionally *Bactrocera neohumeralis* (*Tephritidae*) are found where the larvae for this study were collected. Adult *B. neohumeralis* can be morphologically distinguished from adult *B. tryoni* [47]; therefore, larvae from collected peaches not used in the dissections were reared out to adults for identification. In addition, the mitochondrial cytochrome c oxidase subunit I (*COI*) gene was sequenced from DNA extracted from the larval remains after dissection, as described below, to ensure each larva belonged to the *B. tryoni* species complex. To identify differences between gut bacterial communities of wild and domesticated *B. tryoni* larvae, laboratory-reared *B. tryoni* colonies were sourced from Gosford Primary Industries Institute (GPII.Col) and Macquarie University (MQ.Col) and mass-reared larvae from the Elizabeth Macarthur Agricultural Institute (EMAI) Fruit Fly Production Facility (FFPF.Col). Both the MQ colony and FFPF colony originated from the GPII colony, which was reared out of infested feijoas collected from Gosford (NSW, Australia) in March 2012. Although originating from the same colony, the larvae were from different generations when dissected, reared for a duration in a different laboratory or facility, and fed different batches of diet, and the EMAI FFPF colony was mass reared. Consequently, each domestic colony analyzed in this study would have been exposed to different environmental microbes. The GPII and MQ colonies were always reared on carrot diet consisting of 0.338 L dehydrated carrot, 2.5 g sodium benzoate, 9 g citric acid, and 60 g Torula yeast per liter of water. The FFPF colony was mass reared on a lucerne chaff diet (144 L lucerne chaff, 5.6 kg Torula yeast, 11.3 kg sugar, approximately 99 L water, 307 g sodium benzoate, 250 g methyl paraben, 588 g citric acid; for 27 trays), and then a subset of flies at F54-F57 was transferred back to the larval carrot diet (recipe as above) for five generations prior to dissection and analysis in this study. The adult flies were given continuous access to hydrolyzed yeast, sugar, and water, ad libitum.

**Table 1:** *Bactrocera tryoni* larval populations used in this study

Suffix used in this study	Wild or domesticated (generation)	Substrate	Source	Sampling date^d^	Number of larvae analyzed by NGS
Bux.P	Wild	Peach	Residential property, Buxton, NSW	Jan 2015	20 (from 7 fruit)
Tum.P	Wild	Peach	Residential property, Tumut, NSW	Feb 2015	15 (from 5 fruit)
GPII.Col^a^	Domesticated (F24)	Artificial carrot diet	Gosford Primary Industries Institute, Ourimbah, NSW	Dec 2014	9
FFPF.Col^a,b^	Domesticated (F78-81)	Artificial carrot diet	Fruit Fly Production Facility, Menangle, NSW	Dec 2015	8
MQ.Col^a^	Domesticated (unknown)^c^	Artificial carrot diet	Macquarie University, North Ryde, NSW	June 2015	6

^a^All of these colonies were derived from the same source colony but were maintained at different locations

^b^This colony was reared on lucerne chaff larval diet for at least 40 generations

^c^The generation of this colony is unknown, except it is greater than 24 generations

^d^Sampling date for both domesticated and wild larvae

Individual larvae were analyzed to observe diversity within and between populations. Larvae that had signs of parasitization by parasitoids were discarded. Two or three actively feeding, late second and third instar larvae from each individual fruit were surface sterilized and dissected as previously described [48]. Midguts (from the end of the esophagus, i.e., cardia to the pyloric valve at the start of the hindgut) were used for DNA extraction since we were also interested in finding probiotic candidates that would survive within the artificial carrot larval diet (pH of 3-4.5). Each midgut was homogenized in sterile phosphate buffered saline (PBS) and mixed 1:1 with sterile brain heart infusion broth (BHI-B) containing 40% (*v*/*v*) glycerol and stored at − 80 °C.

For molecular confirmation of wild larval species identity, genomic DNA was extracted from the larval remains, after dissection and midgut removal, using the QIAGEN DNeasy Blood and Tissue Kit. The *COI* gene was PCR amplified using MyTaq™ HS Mix (Bioline), 400 nM of the primers LCO1490 and HC02198 [49], and thermocycling conditions of 95 °C for 2 min, followed by 35 cycles of 95 °C for 15 s, 50 °C for 30 s, and 72 °C for 45 s, and 5 min at 72 °C. Purified amplicons were sequenced using the LCO1490 primer by the Australian Genome Research Facility (AGRF; GenBank accession numbers KY432531-KY432567). The sequences were trimmed using Geneious v. 9.1.14 (https://www.geneious.com/) [50] and compared against the National Center for Biotechnology Information (NCBI) nonredundant nucleotide collection using the MegaBLAST algorithm [51] available on NCBI’s BLAST web interface [52].

### Larval midgut DNA extraction

Midguts were centrifuged at 16,000 × *g* for 5 min to pellet the gut contents and aspirate the BHI-B containing glycerol. The remaining pellet was resuspended in 180 μl of lysozyme lysis buffer [20 mM Tris-HCl (pH 8.0), 2 mM sodium EDTA, 1.2 Triton® X-100, 20 mg/ml lysozyme], vortexed briefly, and incubated at 37 °C for 1.5 h, with a second vortexing at approximately 45 min. DNA was extracted using the MO BIO PowerFecal® DNA Isolation Kit (MO BIO Laboratories) with the following modifications: 570 μl Bead Solution was added to the resuspended midgut and then transferred to a Dry Bead Tube. After the addition of Solution C1, the tube was heated at 65 °C for 10 min, homogenized in a FastPrep® FP120 cell disrupter (MP Biomedicals) for 45 s at a setting of 5.0, and then incubated at room temperature for 10 min. Solutions C2 and C3 (100 μl of each) were added to the sample together instead of in separate steps. To elute the DNA, 50 μl of molecular grade water (Sigma-Aldrich) prewarmed to 50 °C was added to the column, incubated for 5 min at 50 °C, and then centrifuged at 13,000 x *g* for 1 min. The process was repeated using the previous elution. Extracted DNA concentration was determined using the Invitrogen™ Qubit® dsDNA High Sensitivity (HS) Assay Kit (Life Technologies). DNA extraction controls included DNA extractions of the batch of PBS used in the larval dissections and of a couple of colonies of *Lactobacillus plantarum* and *Escherichia coli* cultures. Extracted DNA was stored at − 20 °C.

### Library preparation

In order to improve the taxonomic classification of gut bacterial species, we employed a new NGS method [45, 53]. Burke and Darling [45] provide further detail on the method that we applied and how it compares to conventional V4 region 16S rRNA gene amplicon NGS on an Illumina platform. The 16S rRNA gene library was prepared following the method described by Burke and Darling [45], with the following minor modifications. In brief, each 16S rRNA gene molecule was uniquely tagged with 10 nt random sequences on both ends, fragmented, and pools of fragmented and full-length amplicons sequenced, with the random sequences from either end used to reconstruct the original sequences. Tagging occurred in two steps using polyacrylamide gel electrophoresis (PAGE) purified primers [45] that included a barcode sequence, random tag, and partial Illumina PE adapter sequences in addition to the 27F [54] or 1391R [55] bacterial primer sequences (see Additional file 1). Additional file 1 contains the sequences of all primers used in this study. Each sample had a unique combination of barcodes (see Additional file 2). The first step involved a 50 μl PCR containing 1.25 U *Taq* DNA polymerase (QIAGEN), 1X QIAGEN PCR Buffer, 1X QIAGEN Q-Solution, 250 μM dNTPs, 0.25 μM forward primer (long_forward), and 10 ng *B. tryoni* larval midgut DNA. A single primer extension reaction was carried out in a thermocycler using the conditions 95 °C for 1 min, 50 °C for 2 min, and 72 °C for 3 min. This was followed by an Agencourt AMPure XP (Beckman Coulter) magnetic bead-clean-up using a 0.6 volume of beads to PCR following the methods as per Burke and Darling [45]. Cleaned-size selected DNA was eluted in 35 μl molecular grade water. The second step involved a PCR as before using the reverse primer (long_reverse) and 31 μl of the cleaned-up PCR, followed by another round of Agencourt AMPure XP bead clean-up. 16S rRNA gene molecules with unique tags at both ends were then amplified in a PCR with similar conditions to the primer extension reactions but using 28.5 μl of bead-cleaned DNA and 0.25 μM of each PE_1 and PE_2 primers. PCR cycling conditions were 95 °C for 2 min, followed by 35 cycles of 95 °C for 15 s, 50 °C for 30 s, 72 °C for 2 min, and a final extension of 72 °C for 5 min. PCR products (10 μl) were analyzed on a 1% (*w*/*v*) agarose gel prior to another round of magnetic bead clean-up. As previously described, DNA was eluted in 35 μl molecular grade water. Regardless of whether there was evidence on the gel of a 1.5 kb band, 3 ng of the amplified products was pooled along with 10 μl of three PBS DNA extraction control PCR products (considered as negative controls) and 0.5 ng of *E. coli* PCR product. To ensure there were no traces of primers in the pooled sample, it was subjected to a clean-up using 0.6 volumes of Agencourt SPRIselect Reagent (Beckman Coulter) following the manufacturer’s instructions. The DNA was eluted in 35 μl UltraPure water (Life Technologies), and concentration of the pool was quantified using the Qubit® dsDNA HS Assay Kit.

### Tagmentation of pooled 16S rRNA gene amplicon libraries

Tagmentation, that is, fragmentation of the uniquely tagged, near full-length 16S rRNA gene amplicons using a transposase that simultaneously adds an adapter sequence for use on the Illumina platform, was performed using the Nextera DNA kit following the methods described by Burke and Darling [45], except the Nextera enzyme was diluted 1 in 100 and 1.5 ng of the pooled 16S rDNA amplicon libraries was used as template. The tagmentation reaction was split into two, and two separate PCRs were carried out to produce a pool of DNA fragments with either PE_1 (10 μM) and the Illumina Nextera i7 adapter (N704) or the PE_2 (10 μM) and the Illumina Nextera i5 adapter (S504) at the ends (see Additional file 1 for primer sequences). PCRs consisted of 1X PCR Master Mix from the KAPA HiFi Library Amplification Kit (Kapa Biosystems), N704 and PE_1 primer or S504 and PE_2 primer, 2.5 μl of each primer. PCR cycling conditions are 72 °C for 3 min, 98 °C for 30 s, followed by 12 cycles of, 98 °C for 15 s, 55 °C for 30 s, and 72 °C for 1 min, and then a final extension at 72 °C for 5 min. The tagmentation reactions underwent two rounds of magnetic bead clean-up using 0.6 volumes of SPRIselect beads and were eluted in 35 μl UltraPure water each time. The molarity of the amplified cleaned-up tagmented amplicon libraries and the near full-length pooled amplicon libraries was assessed on an Agilent 2100 Bioanalyzer high-sensitivity DNA chip (Agilent Technologies).

### Sequencing of near full-length and tagmented 16S rRNA gene libraries

Tagged, near full-length 16S rRNA gene amplicons were combined with cleaned tagmented products at a ratio of 1:7 calculated on the molarity of the tagmented products within the range 400–1000 bp. A bioanalyzer was used to determine the amount of product within each size range. A final pool of 3.77 pM (concentration calculated from 400 to 2000 bp range) was spiked with 0.3 pM PhiX and loaded on an Illumina MiSeq flow cell following the Illumina’s MiSeq Reagent Kit v2 Reagent Preparation Guide. Paired end 250 nt reads were sequenced (i.e., 2 × 250 bp; 500 cycles).

### Data analysis

The reconstruction of near full-length 16S rRNA gene sequences from tagged Illumina reads was performed as per the method of Burke and Darling [45]; scripts are available on GitHub in the koadman/longas repository: https://github.com/koadman/longas. Briefly, inside the A5-MiSeq pipeline, the reads first undergo adapter and quality trimming with Trimmomatic [56], then error correction with the SGA k-mer correction algorithm [57]. Next the corrected reads are assembled with the IDBA-UD algorithm [58]. Some templates resulted in two or more assembled sequences (scaffolds 0-4). The script provided in Additional file 3 was used to identify assembled sequences with only a scaffold 0 and no scaffold 1, as it was not realized at this point that there were scaffolds 0 with no scaffold 1, but scaffold(s) 2-4. Scaffold 0 sequences with scaffolds 2-4 were removed when clustering OTUs as they were < 1300 nt, and only 16S rRNA gene sequences > 1300 nt in length were used for the downstream analysis. Scripts, parameters, and mapping files used to analyze the data are provided in Additional files 4, 5, and 6. Sequences were aligned using the align.seqs.py command using the default settings [59]. The alignment was manually trimmed to remove any remaining primer sequences using the AliView alignment editor [60]. Sequences that failed to align were manually checked against the NCBI nonredundant nucleotide collection using MegaBLAST. Gaps were removed from the alignment in AliView.

Near full-length sequences (1300-1500 nt) were analyzed with QIIME 1.9.1 [61] using the Oracle VM VirtualBox version 5.0.26 r108824. Operational taxonomic units (OTUs) were picked using the two-step open-reference picking method using the UCLUST algorithm [62], as implemented in QIIME using the pick_open_reference_otus.py command. Default settings were used, with the exception of picking OTUs at 99% similarity and training the RDP classifier [63, 64] against the Greengenes 16S rRNA gene reference database version 13_5 preclustered at 99% [65]. The default settings included a minimum of two sequences per OTU. Representative OTU sequences (Additional file 7) that clustered with a sequence in the database were named with the number of the sequence in the database, and de novo picked OTUs were given the prefix *de_novo*. As part of the pick_open_reference_otus.py workflow, representative sequences from each OTU were selected and aligned against the Greengenes database gg_13_8 preclustered at 85% identity using PyNAST [59] and used to build a tree using FastTree [66]. The OTU matching to chloroplasts of plant origin (“Streptophyta”) based on Greengenes taxonomic classification was manually removed. To improve taxonomic classification of the OTUs, taxonomic assignment of representative OTU sequences was determined by comparing three different curated databases: the Greengenes database, RDP database (Seqmatch) [63], and NCBI Reference Genomic Sequence database (RefSeq_genomic) [67] and also the NCBI nonredundant nucleotide sequence collection. Representative OTU sequences with the same taxonomic assignment were aligned, and OTUs where the representative sequence differed as a result of degenerate nucleotides that matched the other sequence (nondegenerate nucleotide), or only differed in sequence length, were pooled. Alpha diversity (observed species metric) was computed and rarefaction plots were generated using QIIME for samples with a minimum of 10 and maximum of 260 near full-length sequences with steps of 10, performing 1000 iterations at each step.

To determine whether gut bacterial diversity is the same in wild compared to domestic larvae, a Mann–Whitney U-test was performed on the number of OTUs from each wild and domestic larva. These data were entered into the program at http://www.socscistatistics.com/tests/mannwhitney/Default2.aspx, and a one-tailed test with a significance level of 0.01 was selected.

To determine the level of sequence variation within one OTU, sequences that clustered to the OTU with the greatest amount of near full-length sequences, that is, OTU 808997, were clustered as described above except at 100% similarity with a minimum of one scaffold per OTU. To demonstrate the advantage of working with near full-length sequences, we also clustered the V4 region of the near full-length sequences using the same parameters.

### Culturing of larval midgut microbiota

As a means of validating the results of our high-throughput 16S rRNA gene sequencing survey, we attempted to gain a priori knowledge of the general composition of the culturable microbial community. A selection of larval midguts that were not used for the NGS study, from white-fleshed peaches of the one tree sampled in Buxton or *B. tryoni* domesticated colony, was plated onto a range of agar plates, including de Man, Rogosa, and Sharpe (MRS) agar plates incubated at 37 °C under 5% CO_2_ or anaerobic conditions, or 30 °C in aerobic conditions; Luria-Bertani (LB) agar plates incubated at 37 °C; Tryptone soya agar (TSA); and nutrient agar (NA), yeast dextrose carbonate (YDC) agar, potato dextrose agar (PDA), and glycerol-yeast agar (GLY pH 5) plates incubated at either 25 °C or 30 °C. At least two midgut samples were plated per population, from which a selection of the dominant colonies were subcultured using the same conditions at which colonies were initially isolated and underwent subsequent analyses. No larval midguts were plated from larvae sourced from the peaches from Tumut.

### Bacterial DNA extraction and identification

Isolates were Gram stained and bacterial DNA was extracted using Chelex® 100 molecular biology grade resin (Bio-Rad) from a chosen set of isolates. To determine the molecular identification of the bacterial isolates, the 16S rRNA gene from the extracted DNA was PCR amplified in a 15 μl reaction containing MyTaq™ HS Mix (Bioline), 400 nM 27F [68] and 1492R [69] primers, and 1.5 μl extracted bacterial isolate DNA. PCR cycling conditions used were 95 °C for 2 min, followed by 35 cycles of 95 °C for 30 s, 50 °C for 30 s and 72 °C for 90 s, and a final extension time of 5 min at 72 °C. Purified PCR products were Sanger sequenced by AGRF using just the 27F primer or both the 27F and 1492R primers (GenBank accession numbers KY427098- KY427111). Geneious v.5.3.6 (https://www.geneious.com/) [50] was used for trimming and aligning the forward and reverse sequences. A BLAST search, as described earlier, was performed on the trimmed forward or consensus sequences. Identity was determined by database matches with E values of 0, identity of 99–100% and 100% coverage. The isolate 16S rRNA gene sequences were also aligned using Geneious to respective representative OTU sequences to determine similarity.

## Results

### Identification of wild larvae

Sequencing of the *COI* genes of the 35 wild larvae analyzed indicated that larvae belong to the *B. tryoni* species complex. The only other *B. tryoni* species complex member that may be present, *B. neohumeralis*, was not reared from the infested peaches collected. Only larvae that did not have any obvious signs of parasitoid attack were used in the dissections; however*, B. tryoni* larval parasitoids, *Diachasmimorpha kraussii* and *Diachasmimorpha tryoni*, emerged from peaches from Buxton and Tumut.

### Amplification of bacterial DNA from larval gut samples

Low amounts of DNA were extracted from individual midgut samples (0.24-4.30 ng/μl in a total volume of approximately 40 μl). The amount of amplified tagged bacterial DNA after completing the first three rounds of PCR of the library preparation (5′ end tagging, 3′ end tagging, and then amplification) also differed between samples (see Additional file 8).

### Assembly of full-length reads generated by dual molecular tagging

All of the reads derive from fragments of DNA that underwent dual molecular tagging and amplification. Insufficient depth of sequencing would cause one of two problems: (1) The same molecular tags are not observed more than once—meaning that more 16S rRNA genes were tagged than were sequenced. For the read 1 molecular tags, 80,570 were observed more than once, while only 18,926 were observed once. For the read 2 molecular tags, 95,559 were observed more than once, while 62,799 were observed once. Thus, in both cases, the molecular tag diversity had been comprehensively sampled. The tag counts are shown in Additional file 9, where a bimodal distribution can be observed: a peak at 1 corresponding to tags observed only once, due to sequencing error, and a second broader peak containing tags observed many times, corresponding to true tags. (2) Not enough reads were generated to assemble across each dual-tagged 16S rRNA gene, leading to a low fraction of the tagged genes getting full-length assemblies. In our data, 93.3% of reads map back to the assembled 16S rRNA gene sequences. This suggests that a small fraction (~ 6%) of the molecular tag clusters may have had insufficient data to complete assembly or were reads that did not assemble due to the presence of chimeric PCR products.

A total of 32,337 sequences were assembled based on the molecular tags from 5,160,368 Illumina read pairs. No assembled sequences were obtained for two larval samples (Table 2). As a result of the high number of templates tagged in the positive control, no single tagged template of the positive control had enough paired reads covering the ends of the amplicon to be confidently called as a true template. In the blank DNA extraction controls, only two templates from just one control were successfully tagged and amplified (Table 2). Assembled sequence lengths followed a bimodal distribution with a broad peak around 500 nt and a sharp peak around 1400 nt (histogram shown in Additional file 10). There was one 1711 nt sequence that matched to plant mitochondria. Some templates resulted in two or more assembled sequences (scaffolds 0-4) per tag cluster. Manual inspection indicated these were likely the result of molecular tag collisions, as discussed in Burke and Darling [45]. Typically scaffold 1-4 assembled sequences were < 1300 nt. Approximately 45.5% of the total number of assembled sequences was > 1300 nt. Complete assemblies of the near full-length 16S rRNA gene were defined as having a scaffold 0 with no matching scaffold 1-4 and a length of 1300–1500 nt. To increase the accuracy of taxonomic classification and phylogenetic resolution, only complete assemblies were analyzed. After aligning and removing primer sequences, 14,710 near full-length 16S rRNA gene sequences were included in the OTU clustering, with a differing number of near full-length 16S rRNA gene sequences obtained between samples (Table 2). Near full-length sequences were obtained for 56 larval samples and one PBS blank DNA extraction control.

**Table 2:** Number of near full-length 16S rRNA gene sequences per larval midgut and 99% OTUs detected

Wild or domesticated	Location; host/diet	*Bactrocera tryoni* larval ID	Near-full length sequences (> 1300 nt)	OTUs at 99% similarity^a^
Wild	Buxton; Peach A	Bux.P.A1	193	3
		Bux.P.A2	329	4
		Bux.P.A3	468	3
	Buxton; Peach B	Bux.P.B1	787	8
		Bux.P.B2	349	8
		Bux.P.B3	59	2
	Buxton; Peach C	Bux.P.C1	6	1
		Bux.P.C2	0	0
		Bux.P.C3	18	1
	Buxton; Peach D	Bux.P.D1	3	1
		Bux.P.D2	33	4
	Buxton; Peach E	Bux.P.E1	9	1
		Bux.P.E2	2	1
		Bux.P.E3	628	2
	Buxton; Peach F	Bux.P.F1	425	4
		Bux.P.F2	852	3
		Bux.P.F3	639	5
	Buxton; Peach G	Bux.P.G1	190	9
		Bux.P.G2	830	7
		Bux.P.G3	61	5
Wild	Tumut; Peach A	Tum.P.A1	592	9
		Tum.P.A2	121	4
		Tum.P.A3	45	2
	Tumut; Peach B	Tum.P.B1	322	4
		Tum.P.B2	362	2
		Tum.P.B3	371	2
	Tumut; Peach C	Tum.P.C1	211	8
		Tum.P.C2	50	4
		Tum.P.C3	0	0
	Tumut; Peach D	Tum.P.D1	528	3
		Tum.P.D2	126	3
		Tum.P.D3	949	13
	Tumut; Peach E	Tum.P.E1	520	8
		Tum.P.E2	178	7
		Tum.P.E3	360	8
Domesticated	EMAI Fruit Fly Production Facility; artificial carrot diet	FFPF.Col.1	86	3
		FFPF.Col.2	12	3
		FFPF.Col.3	15	2
		FFPF.Col.4	16	3
		FFPF.Col.5	12	1
		FFPF.Col.6	12	2
		FFPF.Col.7	57	2
		FFPF.Col.8	596	2
Domesticated	Gosford Primary Industries Institute; artificial carrot diet	GPII.Col.1	557	2
		GPII.Col.2	100	2
		GPII.Col.3	143	4
		GPII.Col.4	98	1
		GPII.Col.5	21	1
		GPII.Col.6	540	2
		GPII.Col.7	21	1
		GPII.Col.8	26	1
		GPII.Col.9	262	3
Domesticated	Macquarie University; artificial carrot diet	MQ.Col.1	155	1
		MQ.Col.2	243	1
		MQ.Col.3	21	1
		MQ.Col.4	76	3
		MQ.Col.5	109	1
		MQ.Col.6	837	3
	Blank controls	PBS 1 PBS 2 PBS 3	2 0 0	1 0 0
	Positive control	*E. coli*	0	0
	Total		14,633	195

^a^Chloroplast OTUs have been excluded

### OTUs from the near full-length sequence data

OTUs were assigned at 99% sequence similarity yielding a total of 40 OTUs, after the OTU matching to plant chloroplast DNA, which was detected in eight wild larval gut samples, was removed (Table 2). Taxon status was assigned to each OTU representative sequence by comparing three different databases. The OTU with the highest number of sequences was assigned to the genus *Swaminathania* using the Greengenes database, but was classified as *Asaia* using the RDP and the NCBI Reference Genomic Sequence databases. *Swaminathania* and *Asaia* are closely related genera within the acetic acid bacteria (AAB) group; however, *Asaia* is a common insect symbiont [70], while *Swaminathania* has mostly been found in association with wild rice [71]. The sequences of two representative OTUs were either identical to other representative OTU sequences apart from their length (in one case a difference of four nucleotides) or degenerate nucleotides (a total of four), thus were considered as the same OTUs and pooled as one in the OTU table. Therefore, final number of OTUs detected in the data set was 38. The breakdown of OTUs detected in each sample is provided in Additional file 11.

### Larval gut bacterial diversity

The median number of OTUs in the wild larvae was four, twice that of domesticated larvae. The number of OTUs in wild versus domesticated larvae was significantly higher (*p* < 0.01) (Mann–Whitney U-test; one-tailed). One larva from Tumut peach D (Tum.P.D3) had the highest number of OTUs (13). Typically, when more than two OTUs were detected in a domesticated larva, the majority of sequences clustered to *Asaia*, and then only one or two sequences clustered to other OTUs, such as *Janibacter*, *Propionibacterium acnes*, and *Methylobacterium*.

**Fig. 1:**
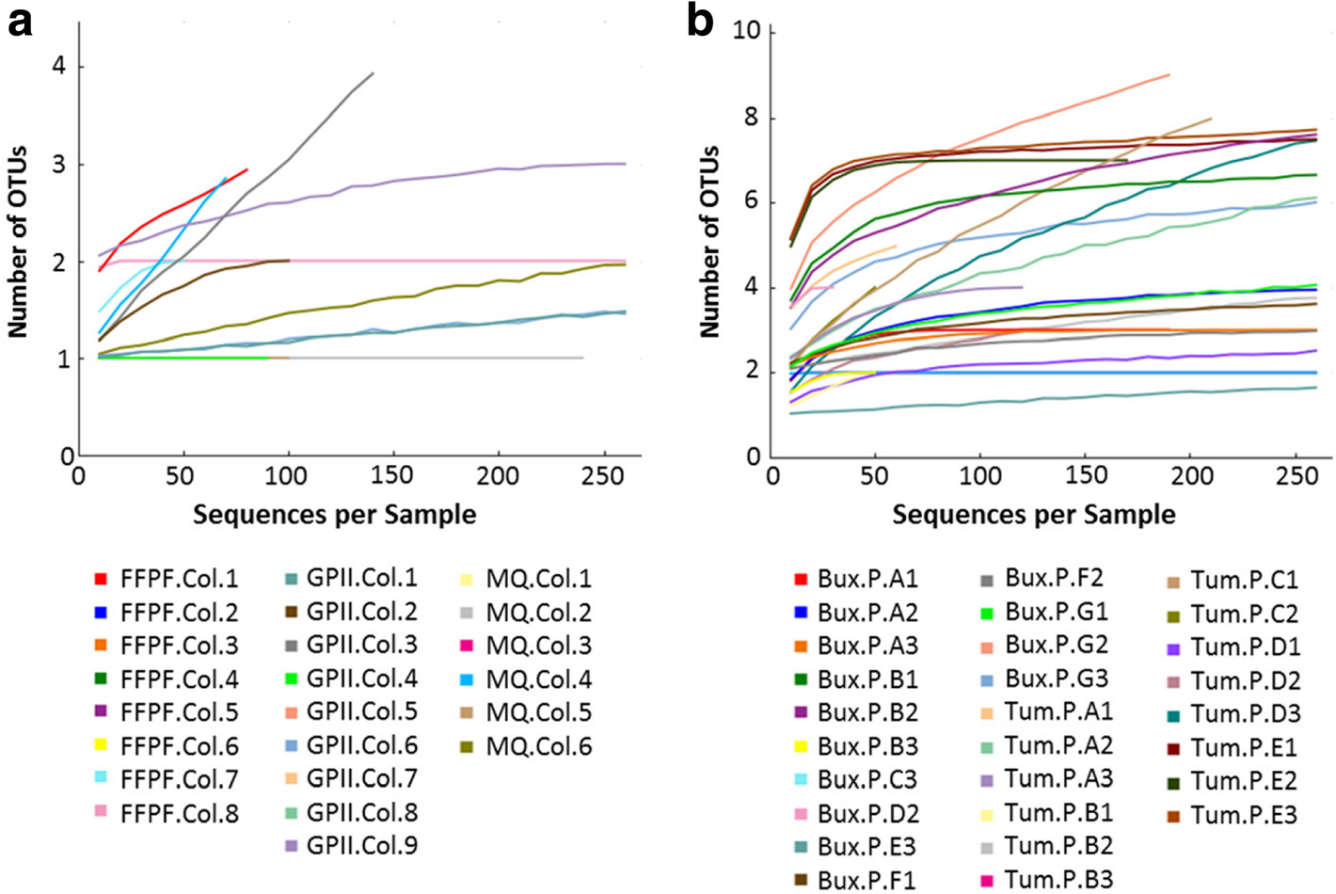
Rarefaction analysis of observed 99% OTUs for midgut bacterial communities of *Bactrocera tryoni* larvae. (**a**) Larval samples from domesticated colonies. (**b**) Larval samples from field-collected peaches. Further details on library identifiers are given in Table 2. Only samples with 10 or more near full-length sequences have been included

The low number of sequences for some samples could indicate that we did not capture the entire bacterial diversity in some samples; however, rarefaction analysis showed that the number of observed species generally did not increase with sequence counts for the majority of domesticated larval gut samples, except for MQ.Col.4, FFPF.Col.1, and GPII.Col.3 (Fig. 1a). Apart from a relatively dominant *Asaia* OTU, the other OTUs in these three samples belong to either the *Halomonadaceae* family or have only one sequence matching to an OTU that only one or two sequences in other samples clustered to. For example, such OTUs include a different (and de novo picked) *Asaia* OTU, *Janibacter, Propionibacterium acnes*, or *Methylobacterium*. In contrast, for a large number of wild larval gut samples, the number of observed species plateaus with increased sequence count (Fig. 1b), suggesting we have captured the majority of the diversity and certainly the most abundant taxa.

**Fig. 2:**
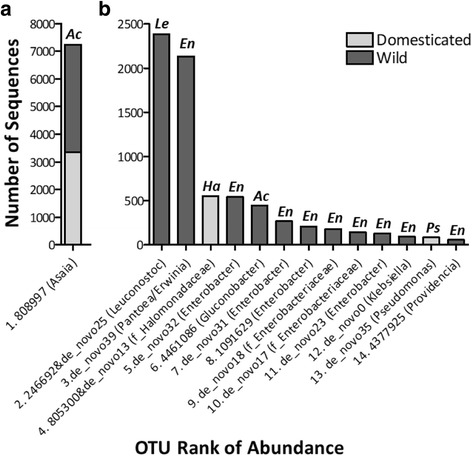
Rank abundance plot of OTUs with more than 50 sequences in *Bactrocera tryoni* larvae sampled. Larvae were sampled from wild or domesticated populations. (**a**) Most abundant OTU with regards to number of sequences. (**b**) Next 2-14 most abundant OTUs. The rank followed by the OTU number (de_novo refers to OTUs from aligning input sequences that failed to find a match to the reference collection), followed by taxonomy assigned to that OTU in brackets, is listed. Where possible taxonomic assignment is at the genus level, otherwise the family level is provided and indicated with an “f” at the start of the name. Above each column are letters to indicate the family that the OTU belongs to (*Ac* = *Acetobacteraceae*; *Le* = *Leuconostocaceae*; *En* = *Enterobacteriaceae*, *Ha* = *Halomonadaceae*; *Ps* = *Pseudomonadaceae*)

Overall, only a few abundant OTUs were detected (Fig. 2). The remaining OTUs were not as common, resulting in a long right-hand tail, which was longer than what is shown in Fig. 2, as only the top 14 out of 38 OTUs are included. The majority of the most abundant OTUs were detected in wild larvae. While *Asaia* is dominant in both wild and domesticated *B. tryoni* populations, very few other OTUs were detected in both wild and domesticated populations (Fig. 2 and Additional file 11).

### Taxonomic composition of the near full-length sequence data

**Fig. 3:**
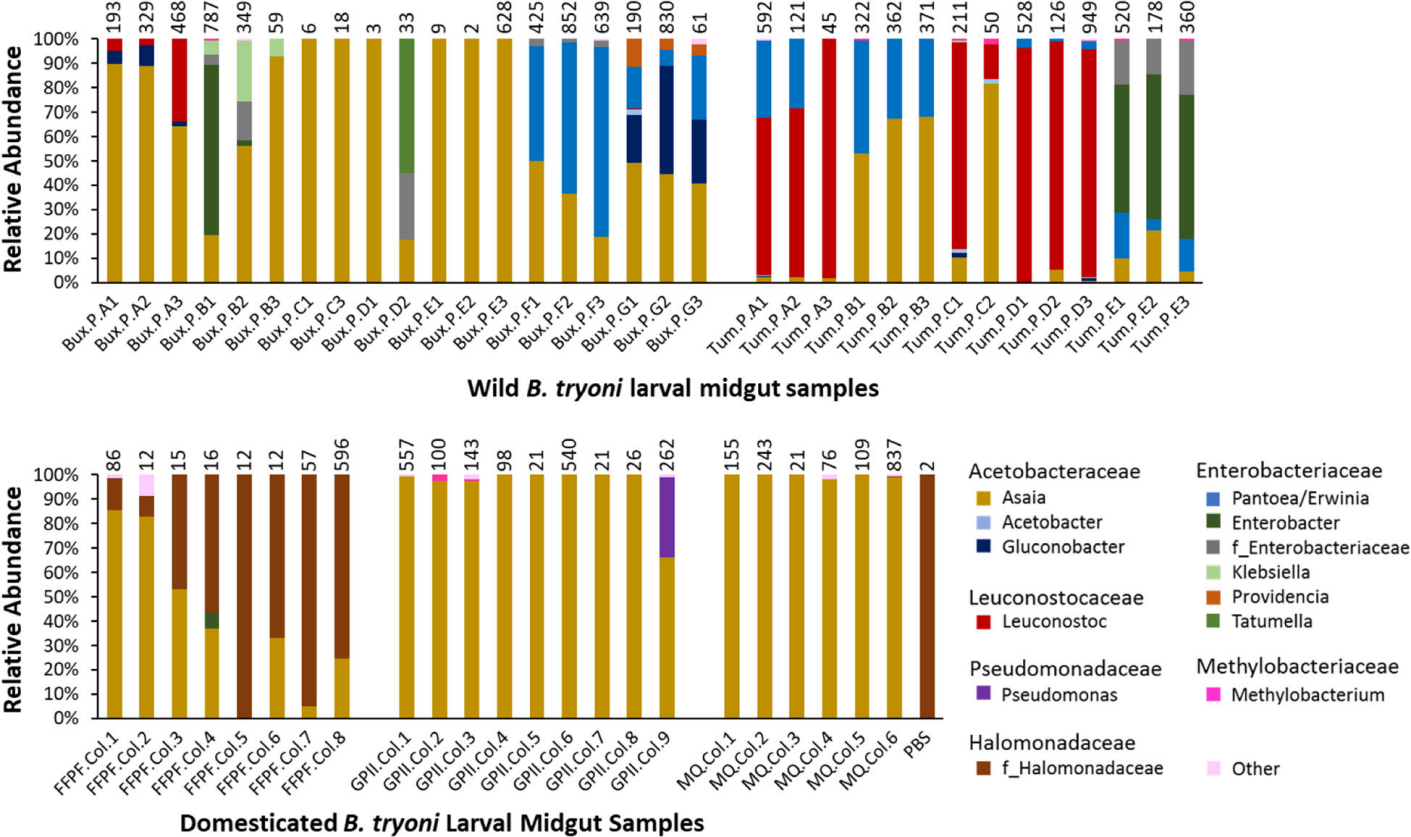
Relative abundance of bacterial taxa in *Bactrocera tryoni* larval midguts. Near full-length sequences were clustered at 99% similarity. Sequences belonging to OTUs from the same genus or family (when genus could not be determined due to the representative OTU sequence matching to 16S rRNA gene sequences from various genera with similar identity) were pooled. The group “Other” includes OTUs with five or less sequences and does not belong to the other families listed. The prefixes Bux, Tum, FFPF, GPII, and MQ refer to the source of samples, and P and Col indicate whether the larva was from a peach or a domesticated colony, respectively. Larvae from the same peach have the same letter before the larval number. For example, Bux.P.A1, Bux.P.A2, and Bux.P.A3 were different larvae from the same whole peach. The number of near full-length sequences included in the OTU clustering for each sample is listed above the respective column

16S rRNA gene sequencing showed the gut bacterial community in *B. tryoni* larvae was predominantly comprised of *Alphaproteobacteria* and *Gammaproteobacteria* (both phylum *Proteobacteria*) and *Bacilli* (phylum *Firmicutes*). Four bacterial families were predominant: *Acetobacteraceae, Enterobacteriaceae, Halomonadaceae*, and *Leuconostocaceae* (Figs. 2 and 3). Whether the OTU belonging to the *Halomonadaceae* family is a significant species in *B. tryoni* gut samples is questionable, as the PBS DNA extraction control possessed the same OTU.

The analysis of individual *B. tryoni* larvae identified that *Asaia* was commonly detected in all populations. The *Asaia* OTU 808997 was detected in 55 out of 56 *B. tryoni* larval gut samples. This OTU was relatively abundant in the majority of the domesticated larvae and wild larvae from Buxton; however, the relative abundance of *Asaia* was lower in the majority of the larvae from Tumut. *Asaia* was the only AAB found in the domesticated larvae, while *Acetobacter* and *Gluconobacter* were also detected in the wild larvae.

In contrast to the laboratory larvae reared on carrot diet, *Asaia* was only relatively dominant in three of the eight mass-reared larvae (from FFPF) that were previously reared on lucerne chaff diet; *Halomonadaceae* was the dominant bacterial family in the other five samples. The identity of this OTU at the genus level is unclear as it aligns with various genera within this family. Since the two near full-length sequences obtained for one of the PBS extraction controls also clustered to the same *Halomonadaceae* OTU found in the FFPF and one larva from the MQ colony (MQ.Col.6), it is possible that the *Halomonadaceae* OTU is a contaminant. Overall very low numbers of near full-length sequences were obtained for the majority of the FFPF gut larval samples; however, in one FFPF larval sample, 596 near full-length sequences were obtained of which 75% of sequences clustered to a *Halomonadaceae* OTU. Apart from *Asaia* and the *Halomonadaceae* OTUs, the only other OTU with more than two near full-length sequences per sample detected in domesticated larvae was *Pseudomonas* (*de_novo*35, near full-length sequences = 85 and only detected in one larva). *Pseudomonas* was not detected in any of the wild larvae analyzed.

*Enterobacteriaceae* were detected in 22 out of 33 wild larval samples, but only detected in one larval sample from a domesticated fruit fly colony (FFPF.Col.4). Working with near full-length 16S rRNA gene sequences improved the classification of some *Enterobacteriaceae* OTUs to the genus level; however, a small number remained for which we could not determine the genus as they matched to a range of *Enterobacteriaceae* genera or unclassified *Enterobacteriaceae* (see Additional file 11). These were assigned to the group “f_Enterobacteriaceae” (Fig. 3). Apart from this group, genera within *Enterobacteriaceae* that were detected include *Enterobacter*, *Klebsiella*, *Providencia,* and *Pantoea/Erwinia*, with similar genera detected in larvae feeding on the same whole fruit. For example, *Providencia* were detected in three larvae, all from the same peach (Buxton peach G), but were not detected in any other larvae. Both *Pantoea* and *Erwinia* species were among the best matches for the OTUs 538677 and *de_novo*39, and since taxonomic revisions have recently occurred within these genera, we chose to refer to these OTUs as *Pantoea/Erwinia. Enterobacteriaceae* were not present in larvae from Buxton peaches A, C, and E and larvae Bux.P.D1, Tum.P.A3, and Tum.P.C2. Apart from Buxton peach A and Bux.P.E3 larvae, we obtained 50 or less near full-length sequences for these larvae.

Gram-negative bacteria had the highest relative abundance in the majority of the gut samples included in this study; however, in seven wild larval samples, Gram-positive bacteria of the genus *Leuconostoc* (OTUs 246692 and *de_novo*25) were relatively dominant; particularly in larvae from Tumut peaches and notably absent from the domesticated larvae (Fig. 3).

Furthermore, it also revealed that larvae feeding on the same diet within a particular location shared a similar gut bacterial profile (Fig. 3). Despite varying location, rearing conditions, and batches of diet between domesticated colonies, bacterial diversity in domesticated *B. tryoni* larvae analyzed was very similar, excluding the presence of *Halomonadaceae* sequences in the FFPF colony. Wild larvae had a more diverse gut bacterial community. Larvae feeding within the same individual fruit generally possessed a similar bacterial community; however, this was not always representative of the bacteria found in larvae from fruit collected from the same tree (Fig. 3). For example, *Leuconostoc* was relatively dominant in larvae from Tumut peach D, while *Enterobacteriaceae* were dominant in larvae from Tumut peach E. Lower bacterial diversity was observed in larvae from Buxton peaches C and E, which also had a low number of near full-length sequences.

Rarefying our data for beta diversity analyses would exclude a large number of sequences due to the variable number of sequences we obtained between samples (Table 2) and would also reduce the statistical power of the analyses; therefore, we did not perform beta diversity analyses. However, similarities and differences among gut bacterial communities of wild and domesticated populations are evident from Fig. 3.

### Analysis of sequences that clustered to the dominant *Asaia* OTU

**Fig. 4:**
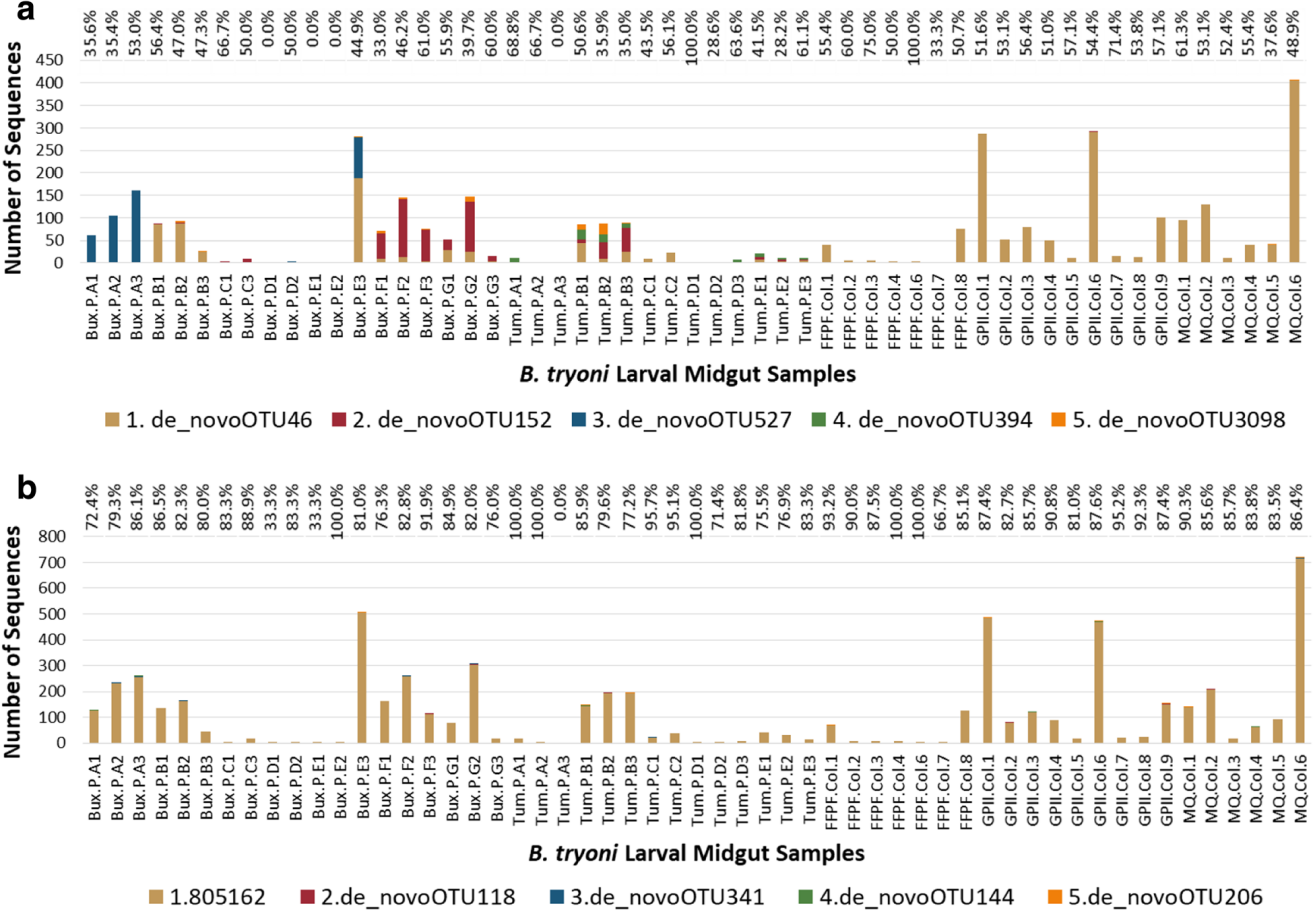
Distribution of near full-length (A) and V4 region (B) top unique *Asaia* (100% similarity) OTUs. The prefixes Bux, Tum, FFPF, GPII, and MQ refer to the source of the samples; P and Col indicate whether the *Bactrocera tryoni* larva was from a peach or a domesticated colony, respectively. Larvae from the same peach have the same letter before the larval number. For example, Bux.P.A1, Bux.P.A2, and Bux.P.A3 represent different larvae from the same whole fruit. The percentage above each column is the respective number of *Asaia* sequences included in the top five unique OTUs of the total number of dominant *Asaia* (99% similarity OTU 808997) sequences for that sample

*Asaia* near full-length 16S rRNA gene sequences that clustered at 99% to the dominant *Asaia* OTU (OTU 808997) were clustered further into groups of unique sequences (e.g., 100% similarity OTUs) and showed greater sequence variation compared to unique sequences from the V4 region only (Fig. 4). The near full-length sequences revealed additional fine-scale diversity of *Asaia* in wild *B. tryoni* larvae, but not in domesticated larvae. The same unique *Asaia* sequences were generally common among larvae from the same peach, or from the same domesticated colony. Some unique *Asaia* sequences were only common to a few wild larvae from a specific location, for example, the *Asaia* unique sequence (100% similarity OTU) called *de_novo*527 was relatively dominant in larvae from Buxton peach A, otherwise only detected in a couple of other larvae from different Buxton peaches. *Asaia* unique sequence *de_novo*394 was only detected in larvae from Tumut peaches. The variation between unique sequences was often only a single nucleotide change. For the top four clusters of unique sequences, a large number of sequences from different samples clustered together; thus, it is unlikely that the variation seen here was due to sequencing errors. The sequence variation observed in some other cases was due to ambiguous nucleotides (Tables 3 and 4). The scripts used to extract the V4 region of these sequences changed ambiguous nucleotides to Ns, and any differences observed among the top five unique V4 sequences were due to the presence of Ns in this region.

**Table 3:** Nucleotide differences between near full-length *Asaia* top five unique (100% similarity) OTU representative sequences

		Position	93	191	766
Unique representative OTU	Number of sequences	Consensus^a^	A	C	A
*de_novo*46	2361				
*de_novo*152	529				G
*de_novo*527	421			T	
*de_novo*394	82		G	T	
*de_novo*3098	68				R

^a^Generated by aligning the top five unique sequence clusters from dominant *Asaia* OTU (clustered at 99%)

**Table 4:** Nucleotide differences between *Asaia* V4 region top five unique (100% similarity) OTU representative sequences

		Position	149	162	199	254
Unique representative OTU	Number of sequences	Consensus^a^	A	A	A	A
805162	6036					
*de_novo*118	12		N			
*de_novo*341	12				N	
*de_novo*144	11			N		
*de_novo*206	11					N

^a^Generated by aligning the representative sequences of the top unique sequence clusters using only the V4 regions of the near full-length 16S rRNA gene sequences of the dominant *Asaia* OTU (clustered at 99%)

### Culturing of *B. tryoni* larval midgut bacteria

Typically, only one morphology type was observed in colonies on agar plates from gut samples of domesticated fruit fly larvae, while several different morphological colony types were often obtained from gut samples of larvae found in the peaches from Buxton. Acid producing bacteria, as indicated by the zone of clearance in the YDC agar around colonies (data not shown), were isolated from all *B. tryoni* larval midguts cultured. Sequencing of the 16S rRNA gene from a selection of such isolates cultured from the FFPF larval midguts revealed that they were *Asaia* sp. (Table 5), and all 16S rRNA gene sequences were identical (data not shown). Isolates of dominant colony types that were sequenced from midguts of larvae collected from peaches in Buxton included *Leuconostoc* and *Enterobacter* species (Table 5). The 16S rRNA gene sequences of the *Leuconostoc* and *Asaia* isolates aligned with no mismatches, apart from sequence length, to the representative OTUs 246692 (*Leuconostoc*) and 808997 (*Asaia*), respectively. The sequences of the *Enterobacter* isolates had occasional nucleotide variation at the same points as the four representative OTUs (1091629, *de_novo*23, *de_novo*31, and *de_novo*32), which could indicate different *Enterobacter* species or variation within copies of the 16S rRNA gene within the species where degeneracies were detected.

**Table 5:** Selection of *Bactrocera tryoni* larval midgut isolates cultured and identified by 16S rRNA gene sequencing

Isolate	*B. tryoni* larva number	Source (location)^a^	Culturing conditions^b^	Isolate ID
126	L112	White-fleshed peach (Buxton)	LB O_2_ 37 °C	*Enterobacter* sp.
132	L117	White-fleshed peach (Buxton)	MRS 5% CO_2_ 37 °C	*Leuconostoc* sp.
133	L117	White-fleshed peach (Buxton)	MRS 5% CO_2_ 37 °C	*Leuconostoc* sp.
136	L117	White-fleshed peach (Buxton)	NA O_2_ 25 °C	*Enterobacter* sp.
155	L117	White-fleshed peach (Buxton)	MRS AnO_2_ 37 °C	*Leuconostoc* sp.
175	L441	FFPF (Menangle)	MRS O_2_ 30 °C	*Asaia* sp.
180	L441	FFPF (Menangle)	TSA O_2_ 30 °C	*Asaia* sp.
182	L441	FFPF (Menangle)	TSA O_2_ 30 °C	*Asaia* sp.
187	L441	FFPF (Menangle)	GLY pH 5 O_2_ 30 °C	*Asaia* sp.
191	L441	FFPF (Menangle)	YDC O_2_ 30 °C	*Asaia* sp.
193	L439	FFPF (Menangle)	TSA O_2_ 30 °C	*Asaia* sp.
197	L439	FFPF (Menangle)	GLY pH 5 O_2_ 30 °C	*Asaia* sp.
198	L439	FFPF (Menangle)	YDC O_2_ 30 °C	*Asaia* sp.
200	L439	FFPF (Menangle)	MRS O_2_ 30 °C	*Asaia* sp.

^a^Fruit Fly Production Facility (FFPF) in New South Wales, Australia

^b^*MRS* de Man, Rogosa and Sharpe agar, *TSA* tryptone soya agar, *YDC* yeast dextrose carbonate agar, *LB* Luria-Bertani agar, *GLY pH 5* glycerol-yeast agar, pH 5, *AnO*_*2*_ anaerobic conditions, *O*_*2*_ aerobic conditions

### Resolving OTU taxonomic assignment to species level

Near full-length 16S rRNA gene sequences enabled taxonomic classification of some OTUs to species level, such as *Acetobacter* representative OTU sequences 1144799 and *de_novo*1 to *Acetobacter orientalis* (greater than 99.8% identity and 100% coverage when compared to the NCBI Reference Genomic Sequence collection). However, often it was not possible to taxonomically classify OTUs to species level as a result of closely related species possessing highly similar 16S rRNA gene sequences. For example, MegaBLAST of the *Leuconostoc* OTUs suggest the most likely species are *Leuconostoc pseudomesenteroides*, *Leuconostoc mesenteroides*, or *Leuconostoc mesenteroides* subsp. *mesenteroides* (> 99.6% identity and 100% coverage). Unfortunately, we were unable to determine the *Asaia* species detected. However, working with greater length sequences enabled the identification of possibly different species or strains within the same genus, as already discussed above for *Asaia* when clustered at 100% similarity. Even with clustering at 99% similarity, it was identified that Tum.P.D3 possessed near full-length sequences clustering to three different *Acetobacter* OTUs. This contributed to the 13 OTUs detected in that sample.

## Discussion

We report the first application of a new near full-length 16S rRNA gene sequencing method to investigate the microbial ecology of a pest insect of economic significance, with the aim to improve mass-rearing for pest management strategies, such as SIT. Our study revealed that gut bacterial diversity is low in wild *B. tryoni* larvae and even lower in domesticated larvae. By analyzing high-quality > 1300 nt 16S rRNA gene sequences of bacteria from individual *B. tryoni* larvae, gut bacterial diversity was explored at a finer scale than in previous tephritid microbiome NGS studies. Even with near full-length 16S rRNA gene sequence data, our ability to determine species was limited by the suitability of the 16S rRNA gene to resolve closely related taxa or availability of reference sequences in taxonomic databases. *Asaia* was identified as a common gut bacterium of both wild and domesticated *B. tryoni* larvae. Analyses of gut bacteria of individual larvae from one single fruit, compared to larvae from other fruit on the same tree or larvae from a different location, and domesticated larvae, suggest an association exists between diet (and its microbial ecosystem) and the *B. tryoni* larval gut bacterial community but also exists with domestication and individual pieces of fruit.

This is the first study to use NGS to analyze the gut microbiome of *B. tryoni* larvae. A previous study using this method showed that the taxonomic profiles were highly concordant with those found using the V4 region of the 16S rRNA gene amplicon sequencing of the same communities, suggesting that this method of analyzing microbial communities is sound. While we did not detect many OTUs in each larva, in the previous study that employed this method, a higher number of OTUs were found, while providing the additional advantage of improved taxonomic assignment. To reduce potential biases, we modified the DNA extraction procedure to maximize bacterial lysis and minimize sample loss between steps and used magnetic beads to clean up PCR products rather than a column approach to minimize DNA loss when preparing the library for sequencing. Fewer amplicons are sequenced by our approach relative to the commonly applied V4 region of the 16S rRNA gene amplicon NGS. This means that very rare taxa are less likely to be captured by our method; however, the presented protocol produces less erroneous reads than data generated by standard short read NGS protocols [45], which can contain a large number of noisy, erroneous sequences [72, 73]. Those noisy sequences are well known to create false OTUs, leading to artificially inflated diversity estimates. High-quality sequences were generated in our study, which is demonstrated by the 16S rRNA gene sequences of the *Leuconostoc* and *Asaia* isolates cultured from the larval gut being identical (apart from sequence length) to the representative OTUs to which most sequences, for these respective taxonomic assignments, clustered.

Low levels of bacterial diversity in larvae have been reported in other field-collected tephritids, including the monophagous *B. oleae*, where at 97% similarity 1-2 OTUs were detected in individual larvae [23], and in another study an average of 5.4 ± 3.4 OTUs (97% similarity) per sample (which included both larvae and adult flies) was reported [14]. In a study of field-collected *C. capitata* larvae that used sample pooling, which can lead to a greater number of OTUs, 7-13 OTUs (97% similarity) were detected [24]. These findings are similar to our results, although different methodologies were employed. Indeed, it could be expected that *B. tryoni* larvae have lower bacterial diversity than adult flies, as they are confined to one environment that would have very little, if any, bacteria present prior to the female fly ovipositing into the fruit. Pyrosequencing of microbial communities of adult *B. tryoni* whole female flies only detected 7-16 OTUs [19], and increased bacterial diversity at the adult stage compared to larval and pupal stages has also been reported in *C. capitata* [5, 24]. Lepidopteran larvae appear to lack a resident microbiome; their gut microbes are considered to be transient as they are in low abundance and predominantly leaf-derived [74]. In contrast to these studies, considerably higher bacterial diversity was reported in *Bactrocera dorsalis* (*Tephritidae*) [75] and *Bactrocera carambolae* (*Tephritidae*) [76] larvae. The difference in OTUs between species could be due to the species themselves or the methodologies used. However, in general larval gut bacterial diversity tends to be less than in adults.

The reduced microbial diversity observed in this study of domesticated *B. tryoni* larvae compared to wild larvae has been observed in other insect species, for example, the gypsy moth (*Lymantria dispar*) [77] and cotton bollworm (*Helicoverpa armigera*) [78]. Reduced gut bacterial diversity, and noticeably the absence of *Enterobacteriaceae* and *Leuconostocaceae* (although not detected consistently across all sampled wild larvae), in domesticated *B. tryoni* larvae could have an impact on the quality and performance of the flies produced for SIT and warrants further study. A reduction in gut microbial diversity may lower microbial colonization resistance, thereby allowing pathogenic microorganisms to establish in the gut [79]. This could influence the health of a domesticated fly colony and in severe cases cause a colony to crash. Numerous tephritid studies have shown that mass-reared flies are often inferior to wild flies in several traits, which includes mating competitiveness [80, 81]. Gut microbiota changes during the domestication process, or as a consequence of irradiation, have not been thoroughly studied in parallel with the effect of the changes on the fly; however, in other studies, antibiotics were added to artificial diets to assess the effects of a reduced microbiome in tephritid flies [82–84] or bacterial supplements (probiotics) were added to tephritid larval or adult diets to evaluate their benefits [17, 20, 21, 85, 86]. These studies indicate that gut microbiota perturbations can have a number of consequences, many of which have not yet been well studied. To understand how to mitigate detrimental microbial changes in mass-rearing, such as through the eco-engineering of diets that encourage “beneficial” microbial communities, the cause and effect of microbial changes requires further investigation.

In the present study, the artificial diet, controlled environmental conditions, and handling of eggs could explain why we observed reduced bacterial diversity in domesticated larvae compared to their wild counterparts. Artificial diets are generally more homogenous than wild food sources and often contain antimicrobials to limit the growth of microbes. This perhaps explains the similar gut bacterial profile of domesticated populations despite being maintained in different locations. The extent of this “streamlining” of gut microbiota in domesticated tephritid larvae has not been demonstrated in other tephritid NGS studies as they have analyzed gut microbiota of pools or few individuals [14, 23, 24, 26, 75, 87]. Microbiota streamlining has previously been observed in adult tephritids when the microbiome of pools of different laboratory-adapted tephritid fly populations were compared with field populations [19].

Our microbiome survey is the first to assess the gut microbiota of individual tephritid larvae feeding on the same single fruit, in comparison to larvae from other fruit on the same tree. Gut microbiota variation was high between larvae from different fruit, while larvae from the same single fruit had similar gut microbial profiles. This suggests that larval gut bacteria are largely obtained from the microbes present (the microbial ecosystem) in the habitat and food consumed and larvae most likely assist in spreading the bacteria throughout the fruit/diet and to other larvae.

The fruit microbial ecosystem is likely to differ between individual fruit as a result of fruit physiology and chance [15], antagonistic interactions between microbes and larval density [88], environment, and natural variation in organisms present on the fruit surface. This study would also have detected the set of bacteria introduced into the fruit during oviposition by the female adult fly [5, 24, 28, 29, 31]. Female tephritid fruit flies predominantly introduce bacteria of the *Enterobacteriaceae* family into the fruit during oviposition, which are thought to be important for the larvae [5, 25, 28, 29]. In *B. tryoni*, gut bacteria can be transmitted via ovipositing into areas where *B. tryoni* previously regurgitated [31]; *B. tryoni* regurgitant contains a large number of *Enterobacteriaceae* that also colonize the adult alimentary canal [89]. The succession of bacteria in the fruit may also impact the relative abundance of particular bacteria in the gut of wild *B. tryoni* larvae. For example, *Leuconostoc mesenteroides* is usually present in the early stages of fermentation, which increases the acidity of the fruit and reduces the pH, which favors lactic acid bacteria over *Enterobacteriaceae* [90, 91].

Since larvae feeding within the same single fruit share similar gut profiles, each larva from the same single fruit could also be regarded as a pseudo-replicate with regard to the impact of an individual fruit, while larvae from different fruits are biological replicates. In addition, the number of near full-length 16S rRNA gene sequences for one larva was often similar to the number of sequences from other larvae from the same single whole fruit or colony, thus providing some measure of method reproducibility. Consequently, the uneven sampling depth may, in part, be due to true biological variation in bacterial biomass in the larval guts.

Yeasts are commonly found in fruits [92]. In the present study, *Saccharomyces cerevisiae* was cultured from the midgut of a larva collected from a peach from Buxton (data not shown). Similarly, yeasts were isolated from the midguts of wild *B. tryoni* larvae collected from a range of fruits [48, 93]. Yeasts create an environment that encourages acid-tolerant bacteria, such as *Acetobacteraceae*, as a result of the ethanol they produce [16]. Live yeasts also possess antimicrobial properties and are antagonistic to different types of bacteria [94] but also may provide a source of nutrients promoting the growth of certain bacteria [95]; therefore, they could influence the structure of bacterial populations in fruits and in the larval gut.

Few bacterial families were predominant in the larval midguts analyzed and included *Acetobacteraceae*, *Enterobacteriaceae*, and *Leuconostocaceae* (primarily *Leuconostoc*), which are frequently isolated from fruits [5, 70, 91]. While there are only a few tephritid studies that have looked at the gut microbes of larvae, bacteria belonging to the families mentioned above were reported in *C. capitata* [24] and *B. oleae* [14, 22, 23]. Even at the genus level, only a small selection of genera within each family were detected in the *B. tryoni* larvae analyzed in this study. Correlating with our NGS results, bacteria belonging to each of these families were also cultured from *B. tryoni* larval midguts. In contrast to our results, *Enterobacteriaceae* are present in mass-reared *C. capitata* Vienna 8 GSS larvae [20], and bacteria of the genus *Asaia* have not been reported as common tephritid symbionts.

In previous studies, *Asaia* was detected by 16S rRNA gene sequencing of bacteria within the esophageal bulb of a single adult olive fly, where only “*Ca*. E. dacicola” was found in the other six olive flies analyzed [29]. *Asaia* was also detected in the midgut of an adult olive fly; however, another AAB, *A. tropicalis*, was reported as a common larval and adult symbiont of olive flies found in both wild and laboratory-adapted populations studied over a two-year period [22]. Interestingly, recent NGS studies of wild and domesticated olive flies at various life stages did not detect *Acetobacter* or any other AAB [14, 23]. In the pyrosequencing study of whole *B. tryoni* adult female fly microbiota by Morrow et al. [19], *Asaia* was detected at a relatively low level in one of the three pools of domesticated *B. tryoni* adults analyzed and not in the pool of wild *B. tryoni* adults analyzed. The domesticated *B. tryoni* populations in the study by Morrow et al. [19] were also maintained on a larval carrot diet but contained an additional antimicrobial (methylparaben), which could influence whether *Asaia* is present. Methylparaben was in the lucerne chaff diet used at the FFPF; however, the FFPF flies used in this study were reared on carrot diet without methylparaben for five generations prior to being dissected, but this may have contributed to the different relative amounts of *Asaia* in the FFPF compared to the other two domesticated populations. There were no reports of *Asaia* in culture-dependent isolation studies of adult *B. tryoni* gut microbiota [27, 32, 96–98]. While the use of different methodologies for microbial characterization and sections of the fly studied can influence what bacteria are cultured or detected, it is possible that *Asaia* is maintained at low levels in *B. tryoni* during the adult stage, but are able to thrive during the larval stage by their ability to survive both within the fruit host, artificial larval diet, and the larval gut. The gut microbes present may also be related to fly age [99].

The presence of the genus *Asaia* in the majority of larval samples analyzed suggests that *Asaia* is an important bacterium for the larval stage. Although the relative dominance of *Asaia* in some wild samples was very low, other studies have shown that potential core microbiota of species may have < 0.5% relative abundance in some individuals [100]. The relative abundance of *Asaia* in the domesticated larvae indicates that bacteria of this genus are highly tolerant of components of the carrot diet used in this study, while other microbiota, such as some species within the family *Enterobacteriaceae*, may not. It is also possible that during the domestication and rearing of *B. tryoni* certain characteristics are being selected for, which in turn selects for larvae with particular microbiota. For example, *Asaia* accelerate larval development of the mosquito *Anopheles gambiae* by influencing the expression of host genes involved in cuticle formation [101, 102]. If *Asaia* helps *B. tryoni* to develop faster, it is possible that by selecting for fast-growing larvae during the domestic rearing process, larvae that predominantly contain *Asaia* are being selected for. However, if *Asaia* is being selected for during domestication and domestication reduces the fitness of the flies, it is possible that the increase abundance of *Asaia* may negatively affect fly fitness, which may explain why the abundance of *Asaia* was lower in wild flies.

It is not known whether *Asaia* is vertically transmitted in *B. tryoni*. However, the high relative abundance of *Asaia* in the domesticated larvae and being detected in larvae reared in different laboratories, which are likely to have different environmental microbes, suggests that *Asaia* is probably transmitted both horizontally and vertically. *Asaia* is capable of being spread horizontally among leafhopper *Scaphoideus titanus* by co-feeding and venereal transmission [103] and is found in the gonads and vertically transmitted via egg smearing in *A. gambiae* [104]. *Asaia* has not been reported in *B. tryoni* oviposition sites, on eggs, or in larvae-infested fruit [25, 32, 89], but these studies used bacterial identification methods, such as API 20E, which are designed to identify *Enterobacteriaceae* and not *Acetobacteraceae*. Microorganisms other than *Enterobacteriaceae* were isolated in Fitt and O’Brien [25] and Lloyd et al. [32], but not identified as they were not high in number. In addition, the bacteria present in reproductive organs of tephritids are not well known and have not been studied in *B. tryoni*, but *Asaia* has been detected in the female ovary in *Bactrocera minax* [26]. Since *Asaia* has the ability to invade host tissue of the mosquitoes *Aedes aegypti* and *Anopheles stephensi* after being acquired through the diet [105], there is the possibility that it could colonize other body parts of *B. tryoni* larvae. This, and other environmental factors, could explain why *Asaia* is relatively dominant in the *B. tryoni* larval microbiome.

Acetic acid bacteria are frequently found in insects that have a sugar-based diet [70]; thus, it is not surprising that *Asaia*, along with *Gluconobacter* and *Acetobacter*, were detected in wild *B. tryoni* larvae. Huang and Douglas [106] demonstrated that *A. tropicalis* reduces the glucose content of dietary food, thereby reducing lipid levels of the host *Drosophila*. This is advantageous for any insect feeding on sugar-rich foods, as high sugar intake can reduce feeding and, consequently, lead to a reduction in the acquisition of other limiting nutrients [106]. Acetic acid bacteria are also suited to surviving within the gut of insects, as they are able to tolerate areas of low oxygen [107], and produce a polysaccharide extracellular matrix that may protect the bacteria from the conditions of the gut [105]. Interestingly, defects in pyrroloquinoline quinone-dependent alcohol dehydrogenase (PQQ-ADH) activity of *Acetobacter pomorum* in *Drosophila melanogaster* (*Drosophilidae*), which enhances *Drosophila* insulin/insulin-like growth factor signaling involved in regulating developmental and metabolic homeostasis, can be restored through the addition of the metabolic product of PQQ-ADH, acetic acid, to the diet [108]. The production of acetic acid by *Asaia* may also be part of maintaining *B. tryoni* homeostasis; however, this requires further investigation. *Asaia* sp. also possess the genes necessary for nitrogen fixing [109].

Clustering unique (100% similarity) near full-length sequences from the relatively dominant *Asaia* OTU at 99% similarity and aligning the top five unique OTUs showed that there is some sequence diversity between samples. Although these unique OTU sequences differed by only one or two nucleotides, there were a large number of sequences that clustered to these sequences, and often, when detected, all larvae from the same peach, for instance, had the same unique *Asaia* sequence. This indicates that it is unlikely that sequence differences in the top unique *Asaia* sequences were caused by sequencing errors. There is potential for error in the sequencing process to introduce rare mutations, but these would appear as low abundance unique *Asaia* OTUs. Overall the number of unique *Asaia* OTUs is high, but as they are all de novo OTUs the variation in sequence length and ambiguous nucleotides that arose during the sequencing are likely to have contributed to this. It is also possible that there are multiple copies of the 16S rRNA gene with minor differences; however, these are, in general, not usually more than 1% within the genome [110]. Sequence variation between larvae from different fruit is not as evident when clustering just the V4 region of these sequences, where the same unique *Asaia* OTU dominates the majority of the samples. The near full-length sequences tell us that there is variation in the *Asaia* sequences detected in larvae from different single fruits. Sequence similarities between *Asaia* found in larvae feeding within the same single fruit or artificial diet also indicate that larvae feeding on the same diet share similar gut microbiota. The dominant unique *Asaia* OTU detected in the domesticated larvae was also detected in some wild larvae. Genome sequencing of the dominant *Asaia* species in the domesticated larvae compared to the same *Asaia* species in the wild could reveal if there are significant changes in the species, that is, does fruit fly domestication also domesticate gut associated bacteria? The same dominant unique *Asaia* OTU in the domesticated colonies that have been maintained in different laboratories suggests that this species is successfully maintained within the colony.

Identifying the functional role(s) of the gut bacteria absent in the domesticated larvae but common in the wild larvae is important for understanding the effect of such bacterial loses on mass-reared larvae. *Enterobacteriaceae* are considered to be vertically transmitted by *B. tryoni* and by a number of other tephritids in the wild, suggesting these bacteria are important for larval survival. *Enterobacteriaceae* are recognized as beneficial gut bacteria in *C. capitata* larvae providing metabolic capabilities, such as nitrogen fixing or pectinolysis, to the insect host enabling the insect to adapt to nutrient changes and limitations in their diet (and environment) [5]. The employment of near full-length 16S rRNA gene sequencing enabled better classification of the genera within the *Enterobacteriaceae* family detected. In our study, *Pantoea/Erwinia* was overall relatively abundant; however, the same genera of *Enterobacteriaceae* were not detected in all wild larvae. Thus, it may be possible that various *Enterobacteriaceae* may be able to fulfill the same roles. Apart from the additional role of “*Ca*. E. dacicola” counteracting the host defenses and allowing *B. oleae* larvae to develop within unripe olives [14], and its ability to contribute to nitrogen metabolism [23], specific roles of different *Enterobacteriaceae* in the gut of polyphagous fruit flies remain relatively unresolved. Strains of *Enterobacteriaceae* have been used as both tephritid larval and adult diet supplements, which, in a number of cases, positively influence key fly traits relevant to SIT, such as reduced developmental times, increased pupal weight, and mating competitiveness under laboratory conditions [17, 20, 21, 85, 86].

Very little is known about *Leuconostoc*, a lactic acid bacterium (LAB), in tephritids or even insects. In other tephritid studies, *Leuconostoc* have predominately been isolated or detected in wild larval [24] and adult tephritids [27, 111] or laboratory-reared tephritids that were fed fruit at the larval stage [87]. *Leuconostoc* have also been identified in laboratory-reared *Drosophila* [16] and in low levels in wild populations [16, 112]; however, other LABs, such as *Lactobacillus* spp., are more abundant and have received more attention. *Lactobacillus* spp. provide protection against pathogenic microbes such as *Serratia marcescens* in *Drosophila* [113]. *Leuconostoc* are the most dominant of the LABs found on plants and among the LABs that produce the least amount of acid [114]. Further investigation into the role of *Leuconostoc* in the gut would be worthwhile as certain strains of *Leuconostoc* have human probiotic properties [115, 116], which may be useful in the context of mass-rearing fit flies for SIT.

The presence of *Halomonadaceae* in some FFPF and MQ larvae may be due to contamination. Contamination from the DNA extraction kit and laboratory reagents is not uncommon in studies when the samples have low bacterial biomass [117, 118]. Since the starting amount of DNA extracted from the midgut was relatively low, it would not be unusual for our sequencing results to include contaminants from the molecular reagents. It is possible that bacteria belonging to the *Halomonadaceae* family were present in one of the reagents used in the larval dissections, storage of the FFPF and MQ larval gut samples, or the DNA extraction kit, which were dissected and extracted at a later stage compared to the other larvae. While not detected in our negative extraction controls but detected at very low levels in some samples, *Propionibacterium* and *Janibacter* are *Actinobacteria* that have been sequenced in blank or negative DNA extractions in NGS studies [117]. *Methylobacterium* have also been reported as DNA extraction kit contaminants in NGS datasets [117]. This genus was detected in nine *B. tryoni* gut microbiomes from both domesticated and wild larvae (Additional file 11). If these bacteria are contaminants, the already low number of OTUs detected in the domesticated larvae would be even lower.

## Conclusions

Sequencing of near full-length 16S rRNA gene sequences and comparing individuals facilitated analysis of the composition and diversity of *B. tryoni* larval gut bacteria at a much finer scale than previously possible. The predominant bacteria identified belonged to only three families, suggesting selective factors define what bacteria are present in the gut of *B. tryoni* larvae. In contrast to domesticated larvae, the bacterial communities of wild larvae are more diverse. Higher gut bacterial variation in wild larvae could imply that some bacteria share similar functional roles and can substitute one another; however, variable functions of individual species or strains should also be considered. Gut microbiota can have considerable influence on the host. Thus, the absence of relatively abundant bacterial species found in wild larvae from domesticated larvae highlights that a greater understanding of the functional role(s) of gut microbiota, and the causes and effects of community changes in gut microbiota, is necessary to improve the effectiveness of *B. tryoni* SIT. The novel method used in this study identified important aspects of the *B. tryoni* larval gut community. The accuracy of the method has already been confirmed for more diverse bacterial communities on human skin [45]; however, our study could benefit from parallel evaluation of the method with complementary approaches to further substantiate and expand on our findings.

## Additional files

Additional file 1: Primer sequences. (DOCX 13 kb)

Additional file 2: Primers used to tag 16S molecules. (DOCX 20 kb)

Additional file 3: Code to sort scaffolds. (PY 8 kb)

Additional file 4: Scripts and parameters. (TXT 7 kb)

Additional file 5: Mapping2. (TXT 3 kb)

Additional file 6: Mapping3. (TXT 3 kb)

Additional file 7: Representative OTUs. (FASTA 54 kb)

Additional file 8: Amount of amplified DNA. (XLSX 11 kb)

Additional file 9: Read tag counts. (PDF 4 kb)

Additional file 10: Length distribution of scaffolds. (DOCX 35 kb)

Additional file 11: OTU table. (XLSX 51 kb)

## Abbreviations

AAB: Acetic acid bacterium
AGRF: Australian Genome Research Facility
BHI-B: Brain heart infusion broth
COI: Cytochrome c oxidase subunit I
EMAI: Elizabeth Macarthur Agricultural Institute
FFPF: Fruit Fly Production Facility
GLY: Glycerol-yeast agar
GPII: Gosford Primary Industries Institute
GSS: Genetic sexing strain
HS: High sensitivity
LB: Luria-Bertani
MQ: Macquarie University
MRS: de Man, Rogosa and Sharpe
NA: Nutrient agar
NCBI: National Center for Biotechnology Information
NGS: Next-generation sequencing
OTU: Operational taxonomic unit
PAGE: Polyacrylamide gel electrophoresis
PBS: Phosphate buffered saline
PDA: Potato dextrose agar
PHA: Plant Health Australia
SIT: Sterile insect technique
TSA: Tryptone soya agar
YDC: Yeast dextrose carbonate
