# Supplementary material for: Near full-length 16S rRNA gene next-generation sequencing revealed Asaia as a common midgut bacterium of wild and domesticated Queensland fruit fly larvae
Source: Microbiome. 2018 May 5;6:85. doi: 10.1186/s40168-018-0463-y (PMC5935925; doi:10.1186/s40168-018-0463-y)
Supplement: Supplementary file 1 — Primer sequences. (DOCX 13 kb) [file 40168_2018_463_MOESM1_ESM.docx]

**Additional file 1** Sequences of primers used in this study.

| **Primer name** | **Sequence** |
| --- | --- |
| Long_forward_2 | ACACTCTTTCCCTACACGACGCTCTTCCGATCTNNNNNNNNNNTATTAACTNCGAGAGTTTGATCMTGGCTCAG |
| Long_forward_3 | ACACTCTTTCCCTACACGACGCTCTTCCGATCTNNNNNNNNNNCTAATGGCNNCGAGAGTTTGATCMTGGCTCAG |
| Long_forward_4 | ACACTCTTTCCCTACACGACGCTCTTCCGATCTNNNNNNNNNNAACCAGTCNNNCGAGAGTTTGATCMTGGCTCAG |
| Long_forward_5 | ACACTCTTTCCCTACACGACGCTCTTCCGATCTNNNNNNNNNNGAACGGAGCGAGAGTTTGATCMTGGCTCAG |
| Long_forward_11 | ACACTCTTTCCCTACACGACGCTCTTCCGATCTNNNNNNNNNNTTCAGCGANNCGAGAGTTTGATCMTGGCTCAG |
| Long_forward_12 | ACACTCTTTCCCTACACGACGCTCTTCCGATCTNNNNNNNNNNGGATGCCANNNCGAGAGTTTGATCMTGGCTCAG |
| Long_forward_13 | ACACTCTTTCCCTACACGACGCTCTTCCGATCTNNNNNNNNNNCGGTCGAGCGAGAGTTTGATCMTGGCTCAG |
| Long_forward_14 | ACACTCTTTCCCTACACGACGCTCTTCCGATCTNNNNNNNNNNAAGACTACNCGAGAGTTTGATCMTGGCTCAG |
| Long_forward_15 | ACACTCTTTCCCTACACGACGCTCTTCCGATCTNNNNNNNNNNAACGCTAANNCGAGAGTTTGATCMTGGCTCAG |
| Long_reverse_7 | CTCGGCATTCCTGCTGAACCGCTCTTCCGATCTNNNNNNNNNNTTGGCTATNNNTAGACGGGCGGTGTGTRCA |
| Long_reverse_8 | CTCGGCATTCCTGCTGAACCGCTCTTCCGATCTNNNNNNNNNNTGGCGATTTAGACGGGCGGTGTGTRCA |
| Long_reverse_9 | CTCGGCATTCCTGCTGAACCGCTCTTCCGATCTNNNNNNNNNNCCTCTGATNTAGACGGGCGGTGTGTRCA |
| Long_reverse_10 | CTCGGCATTCCTGCTGAACCGCTCTTCCGATCTNNNNNNNNNNCTCATGCGNNTAGACGGGCGGTGTGTRCA |
| Long_reverse_16 | CTCGGCATTCCTGCTGAACCGCTCTTCCGATCTNNNNNNNNNNGCCTACGCNTAGACGGGCGGTGTGTRCA |
| Long_reverse_17 | CTCGGCATTCCTGCTGAACCGCTCTTCCGATCTNNNNNNNNNNTGACTGCTNNTAGACGGGCGGTGTGTRCA |
| Long_reverse_18 | CTCGGCATTCCTGCTGAACCGCTCTTCCGATCTNNNNNNNNNNATTGCCGCNTAGACGGGCGGTGTGTRCA |
| Long_reverse_19 | CTCGGCATTCCTGCTGAACCGCTCTTCCGATCTNNNNNNNNNNCAACCTTANNTAGACGGGCGGTGTGTRCA |
| Long_reverse_20 | CTCGGCATTCCTGCTGAACCGCTCTTCCGATCTNNNNNNNNNNGGAGGCTGNTAGACGGGCGGTGTGTRCA |
| PE_1 | AATGATACGGCGACCACCGAGATCTACACTCTTTCCCTACACGACG |
| PE_2 | CAAGCAGAAGACGGCATACGAGATCGGTCTCGGCATTCCTGCTGAACCG |
| Illumina Nextera i7 adapter (N704) | CAAGCAGAAGACGGCATACGAGAT**GCTCAGGA**GTCTCGTGGGCTCGG |
| an Illumina Nextera i5 adapter (S504) | AATGATACGGCGACCACCGAGATCTACAC**AGAGTAGA**TCGTCGGCAGCGTC |
| Illumina_E_1 | AATGATACGGCGACCACCGA |
| Illumina_E_2 | CAAGCAGAAGACGGCATACGA |
| 27F | AGAGTTTGATCMTGGCTCAG |
| 1492R | CGGTTACCTTGTTACGACTT |
