# Supplementary material for: Near full-length 16S rRNA gene next-generation sequencing revealed Asaia as a common midgut bacterium of wild and domesticated Queensland fruit fly larvae
Source: Microbiome. 2018 May 5;6:85. doi: 10.1186/s40168-018-0463-y (PMC5935925; doi:10.1186/s40168-018-0463-y)
Supplement: Supplementary file 2 — Primers used to tag 16S molecules. (DOCX 20 kb) [file 40168_2018_463_MOESM2_ESM.docx]

**Additional file 2** List of PAGE purified primers used in this study to uniquely tag each 16S rRNA molecule in each sample. Primer sequences are listed in Additional file 1.

| **Sample Identification** | **Host/Diet** | **Location** | **Step 1 PCR Forward Primer** | **Step 2 PCR Reverse Primer** |
| --- | --- | --- | --- | --- |
| Bux_P_A1 | white-fleshed peach | Buxton | Long_forward_5 | Long_reverse_10 |
| Bux_P_A2 | white-fleshed peach | Buxton | Long_forward_5 | Long_reverse_16 |
| Bux_P_A3 | white-fleshed peach | Buxton | Long_forward_5 | Long_reverse_17 |
| Bux_P_B1 | white-fleshed peach | Buxton | Long_forward_5 | Long_reverse_18 |
| Bux_P_B2 | white-fleshed peach | Buxton | Long_forward_5 | Long_reverse_20 |
| Bux_P_B3 | white-fleshed peach | Buxton | Long_forward_11 | Long_reverse_7 |
| Bux_P_C1 | white-fleshed peach | Buxton | Long_forward_11 | Long_reverse_8 |
| Bux_P_C2 | white-fleshed peach | Buxton | Long_forward_11 | Long_reverse_10 |
| Bux_P_C3 | white-fleshed peach | Buxton | Long_forward_11 | Long_reverse_9 |
| Bux_P_D1 | white-fleshed peach | Buxton | Long_forward_11 | Long_reverse_16 |
| Bux_P_D2 | white-fleshed peach | Buxton | Long_forward_11 | Long_reverse_17 |
| Bux_P_E1 | white-fleshed peach | Buxton | Long_forward_11 | Long_reverse_18 |
| Bux_P_E2 | white-fleshed peach | Buxton | Long_forward_11 | Long_reverse_20 |
| Bux_P_E3 | white-fleshed peach | Buxton | Long_forward_12 | Long_reverse_7 |
| Bux_P_F1 | white-fleshed peach | Buxton | Long_forward_12 | Long_reverse_8 |
| Bux_P_F2 | white-fleshed peach | Buxton | Long_forward_12 | Long_reverse_9 |
| Bux_P_F3 | white-fleshed peach | Buxton | Long_forward_12 | Long_reverse_10 |
| Bux_P_G1 | white-fleshed peach | Buxton | Long_forward_12 | Long_reverse_16 |
| Bux_P_G2 | white-fleshed peach | Buxton | Long_forward_12 | Long_reverse_17 |
| Bux_P_G3 | white fleshed peach | Buxton | Long_forward_12 | Long_reverse_18 |
| Tum_P_A1 | white-fleshed peach | Tumut | Long_forward_13 | Long_reverse_17 |
| Tum_P_A2 | white-fleshed peach | Tumut | Long_forward_13 | Long_reverse_18 |
| Tum_P_A3 | white-fleshed peach | Tumut | Long_forward_13 | Long_reverse_20 |
| Tum_P_B1 | white-fleshed peach | Tumut | Long_forward_14 | Long_reverse_7 |
| Tum_P_B2 | white-fleshed peach | Tumut | Long_forward_14 | Long_reverse_8 |
| Tum_P_B3 | white-fleshed peach | Tumut | Long_forward_14 | Long_reverse_9 |
| Tum_P_C1 | white-fleshed peach | Tumut | Long_forward_12 | Long_reverse_20 |
| Tum_P_C2 | white-fleshed peach | Tumut | Long_forward_13 | Long_reverse_7 |
| Tum_P_C3 | white-fleshed peach | Tumut | Long_forward_13 | Long_reverse_8 |
| Tum_P_D1 | white-fleshed peach | Tumut | Long_forward_13 | Long_reverse_9 |
| Tum_P_D2 | white-fleshed peach | Tumut | Long_forward_13 | Long_reverse_10 |
| Tum_P_D3 | white-fleshed peach | Tumut | Long_forward_13 | Long_reverse_16 |
| Tum_P_E1 | white-fleshed peach | Tumut | Long_forward_14 | Long_reverse_10 |
| Tum_P_E2 | white-fleshed peach | Tumut | Long_forward_14 | Long_reverse_16 |
| Tum_P_E3 | white-fleshed peach | Tumut | Long_forward_14 | Long_reverse_17 |
| FFPF_Col_1 | carrot diet | Menangle | Long_forward_4 | Long_reverse_10 |
| FFPF_Col_2 | carrot diet | Menangle | Long_forward_4 | Long_reverse_16 |
| FFPF_Col_3 | carrot diet | Menangle | Long_forward_4 | Long_reverse_17 |
| FFPF_Col_4 | carrot diet | Menangle | Long_forward_4 | Long_reverse_18 |
| FFPF_Col_5 | carrot diet | Menangle | Long_forward_4 | Long_reverse_20 |
| FFPF_Col_6 | carrot diet | Menangle | Long_forward_5 | Long_reverse_7 |
| FFPF_Col_7 | carrot diet | Menangle | Long_forward_5 | Long_reverse_8 |
| FFPF_Col_8 | carrot diet | Menangle | Long_forward_5 | Long_reverse_9 |
| GPII_Col_1 | carrot diet | Ourimbah | Long_forward_2 | Long_reverse_7 |
| GPII_Col_2 | carrot diet | Ourimbah | Long_forward_2 | Long_reverse_8 |
| GPII_Col_3 | carrot diet | Ourimbah | Long_forward_2 | Long_reverse_9 |
| GPII_Col_4 | carrot diet | Ourimbah | Long_forward_2 | Long_reverse_16 |
| GPII_Col_5 | carrot diet | Ourimbah | Long_forward_2 | Long_reverse_17 |
| GPII_Col_6 | carrot diet | Ourimbah | Long_forward_2 | Long_reverse_18 |
| GPII_Col_7 | carrot diet | Ourimbah | Long_forward_3 | Long_reverse_7 |
| GPII_Col_8 | carrot diet | Ourimbah | Long_forward_3 | Long_reverse_9 |
| GPII_Col_9 | carrot diet | Ourimbah | Long_forward_3 | Long_reverse_10 |
| MQ_Col_1 | carrot diet | North Ryde | Long_forward_3 | Long_reverse_17 |
| MQ_Col_2 | carrot diet | North Ryde | Long_forward_3 | Long_reverse_18 |
| MQ_Col_3 | carrot diet | North Ryde | Long_forward_3 | Long_reverse_20 |
| MQ_Col_4 | carrot diet | North Ryde | Long_forward_4 | Long_reverse_7 |
| MQ_Col_5 | carrot diet | North Ryde | Long_forward_4 | Long_reverse_8 |
| MQ_Col_6 | carrot diet | North Ryde | Long_forward_4 | Long_reverse_9 |
| PBS_Control_1 |  |  | Long_forward_15 | Long_reverse_8 |
| PBS_Control_2 |  |  | Long_forward_14 | Long_reverse_18 |
| PBS_Control_3 |  |  | Long_forward_14 | Long_reverse_20 |
| E. coli_Pos_Control |  |  | Long_forward_15 | Long_reverse_16 |
