# Supplementary figures and images for: Near full-length 16S rRNA gene next-generation sequencing revealed Asaia as a common midgut bacterium of wild and domesticated Queensland fruit fly larvae
Source: Microbiome. 2018 May 5;6:85. doi: 10.1186/s40168-018-0463-y (PMC5935925; doi:10.1186/s40168-018-0463-y)

**read 1 tag counts**

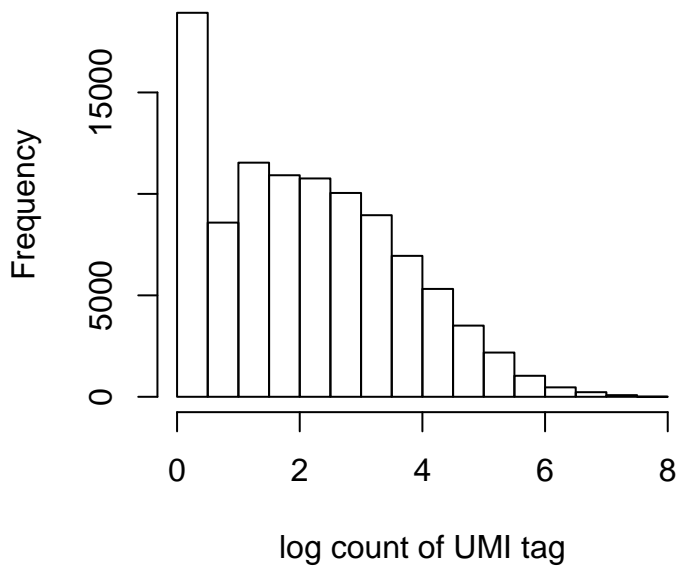

**read 2 tag counts**

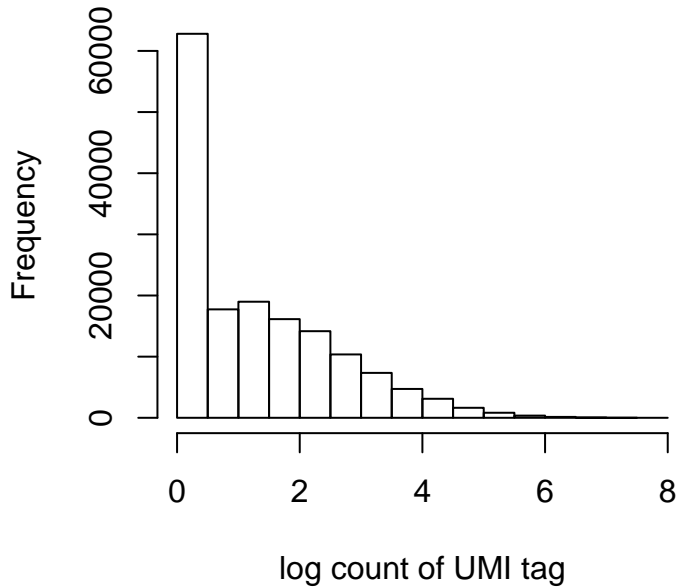

Supplement: Supplementary file 9 — Read tag counts. (PDF 4 kb) [file 40168_2018_463_MOESM9_ESM.pdf]
