# Supplementary material for: Near full-length 16S rRNA gene next-generation sequencing revealed Asaia as a common midgut bacterium of wild and domesticated Queensland fruit fly larvae
Source: Microbiome. 2018 May 5;6:85. doi: 10.1186/s40168-018-0463-y (PMC5935925; doi:10.1186/s40168-018-0463-y)
Supplement: Supplementary file 10 — Length distribution of scaffolds. (DOCX 35 kb) [file 40168_2018_463_MOESM10_ESM.docx]

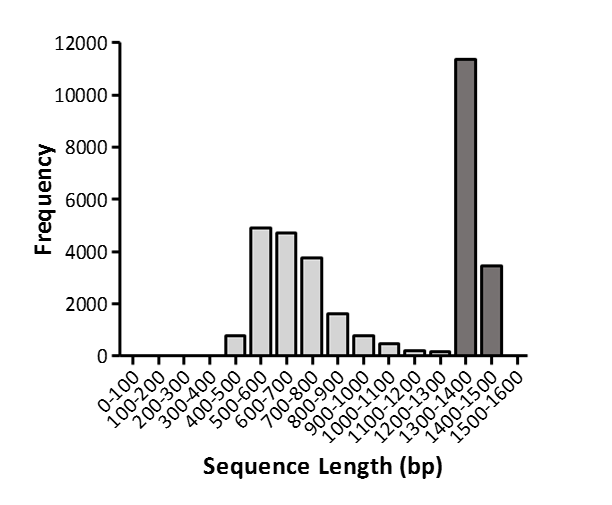


**Additional file 10** Length distributions of assembled sequences, which includes all scaffolds. Shaded in dark grey are the sequence lengths analyzed in this study.
